# Abiotic Stress Response to As and As+Si, Composite Reprogramming of Fruit Metabolites in Tomato Cultivars

**DOI:** 10.3389/fpls.2017.02201

**Published:** 2017-12-22

**Authors:** Marta Marmiroli, Francesca Mussi, Davide Imperiale, Giacomo Lencioni, Nelson Marmiroli

**Affiliations:** Department of Chemistry, Life Sciences and Environmental Sustainability, University of Parma, Parma, Italy

**Keywords:** *Solanum lycopersicum* L., arsenic stress, multivariate analysis, oxidative stress, ripening

## Abstract

The toxic element arsenic interacts with the beneficial element silicon at many levels of the plant metabolism. The ability of the tomato plant to take up and translocate As into its fruit has risen concerns that it could facilitate the entry of this element into the human food chain above the admitted level. Here, the fruit of two contrasting tomato cultivars, Aragon and Gladis, were evaluated following exposures of either 48 h or 14 days to As-contaminated irrigation water, with or without supplementary Si. The focus was on selected biochemical stress response indicators to dissect metabolic fruit reprogramming induced by As and Si. A multivariate statistical approach was utilized to establish the relationship between tissue As and Si concentrations and selected biochemical aspects of the stress response mechanisms to identify a set of relevant stress response descriptors. This resulted in the recognition of strong cultivar and temporal effects on metabolic and biochemical stress parameters following the treatments. In this paper the metabolic changes in H_2_O_2_ content, lipid peroxidation, lycopene and carotenoids content, ascorbate and GSH redox state, total phenolics, ABTS and DPPH radicals inhibition were in favor of an oxidative stress. The significance of some of these parameters as reliable arsenic exposition biomarkers is discussed in the context of the limited knowledge on the As-induced stress response mechanisms at the level of the ripening fruit which presents a distinctive molecular background dissimilar from roots and shoots.

## Introduction

Geologic processes and environmental pollution makes arsenic (As) a ubiquitous metalloid. Volcanic activity is responsible for the release of some 17.15 Mt As annually from the lithosphere (Matschullat, 2000), and anthropogenic activity deposits some 80 Mt worldwide per year into arable soil. One of the most prominent routes by which As reaches cropping soils is the use of As-contaminated water for irrigation (Ng et al., 2003), while the presence of As in some fertilizer and pesticide formulations has been documented to induce significant uptake of As into the edible parts of a range of major crop species (Punshon et al., 2017).

Large-scale regional maps are available for soil arsenic concentrations in Europe (Lado et al., 2008) and United States topsoil (Shacklette and Boerngen, 1984). It is estimated that both developed and developing countries suffer from As contamination, but a global map is not yet available (Punshon et al., 2017; Sarkar and Paul, 2017). The World Health Organization (WHO) estimates that more than 200 million persons worldwide are exposed to concentrations of As in drinking water above the safety standard of 10 μg L^-1^ (Naujokas et al., 2013). A broad range of adverse effects on health, such as cancer, cardiovascular diseases and diabetes, has been associated with human exposure to As (Sharma et al., 2013).

As occurs in natural systems in both organic (as mono or dimethylarsonic acid) and inorganic (arsenate and arsenite) form. It is also known that organic arsenic does account for no more than 10–15% of the total arsenic in food. Whereas, the amount of percentage of organic As in soli depends on type and amounts of sorbing components, pH, and redox potential (Mandal and Suzuki, 2002).

In plants Arsenate is quickly reduced to As(III), which is detoxified by complexation with thiol-rich peptides, such as reduced glutathione (GSH) and phytochelatins (PCs), and/or vacuolar sequestration (Tripathi et al., 2012).

Silicon (Si), the second most abundant constituent of soil and rock, comprises between 0.1 and 10% of plant dry matter. Although this concentration is comparable to that of the essential elements calcium, phosphorus and potassium, it remains controversial whether Si is essential for plant growth and development (Epstein and Bloom, 2005).

The presence of Si has been shown to limit the uptake and root-to-shoot translocation of both arsenite and arsenate in rice seedlings (Guo et al., 2005; Sanglard et al., 2014) and to mitigate the salinity stress in rice, wheat, barley, cucumber and tomato (Al-Aghabary et al., 2004; Pontigo et al., 2015). Si ample stress mitigation capacities are thought to include the facilitation of water uptake by the roots, a reduction in the extent of transpirative water loss and the maintenance of photosynthesis and plant mineral nutrient balance (Zhu and Gong, 2014). In particular Si has been suggested to counter the negative impact of oxidative stress by restricting the production of ROS, enhancing the action of various anti-oxidative compounds and regulating the osmotic potential of the cell (Tripathi et al., 2013; Sanglard et al., 2014). In rice As uptake as arsenite occurs through aquaporine channels shared by Si (Ma et al., 2008) whereas arsenate enters the root via phosphate transporters (Ali et al., 2009). Arsenite (As III) uptake in plants occurs through a subclass of the water channels aquaporin (Panda et al., 2010), whereas arsenate (As V) enters the root via phosphate transporters (Ali et al., 2009).

It has been shown that of these water channels, the specific silicic acid transporters Lsi1 and Lsi2 mediate respectively influx and efflux of silicic acid and arsenite into rice roots (Ma et al., 2008). Therefore it has been suggested that Si fertilization may be a successful procedure for decreasing As accumulation and stress in crop plants, such as rice, grown in As-contaminated soil (Zhao et al., 2009).

Due to the importance of agro-ecosystems in the global environment the co-occurrence of As and Si in soil assumes particular significance. Tomato (*Solanum lycopersicum* L.) fruit is an important component of the human diet worldwide^1^. More than 75,000 accessions are maintained by various ex situ gene banks, and over 7,000 cultivars have been documented (Pesaresi et al., 2014). The genomes of the inbred cultivar ‘Heinz 1706’ and of S. pimpinellifolium LA1589 are available at the Solanaceae Genomics Network website (SGN^2^). Some cultivars respond to As in the soil by concentrating it in the fruit up to a level of 20 ppm dry weight (Burló et al., 1999; Marmiroli et al., 2014). While Si can influence the amount of As translocated to the aerial part of tomato plants, it appears that tomato cultivars differ with respect to their ability to take up and translocate As and Si (Madeira et al., 2012; Kleiber et al., 2015). Among many, the two cultivars (cvs.) ‘Aragon’ and ‘Gladis’ exhibited different fruit morphology and were shown to have markedly different responses to As and Si treatment (Marmiroli et al., 2014).

Both cvs. absorbed and translocated As into the aerial part of the plant but Gladis did it rather inefficiently. Moreover, supplementation with CaSiO_3_ reduced As uptake and translocation in Aragon, while increasing As in Gladis (Marmiroli et al., 2014).

Although ample experimental evidence supports the notion that the presence of As within plant cells induces the production of ROS, it is not clear that oxidative stress is the most important aspect of As toxicity in a ripening fruit undergoing drastic physiological, cellular, and molecular readjustment. Similarly, the role of Si needs to be re-evaluated. The present research was intended to establish whether As evokes metabolic changes as an oxidative stress in the ripening fruit of the two divergent tomato cvs. Aragon and Gladis. Moreover, the efficacy of Si supplementation to alleviate these As-induced changes was assessed. A multivariate statistical analysis was accompanied by factor reduction analysis to establish the degree to which fruit As and Si concentrations were related to selected parameters of stress response, to characterize the interactions between treatment type and time and the fruit ripening process in different cultivars.

## Materials and Methods

### Reagents and Standards

All reagents and standards were purchased from Sigma-Aldrich (St. Louis, MO, United States) unless stated otherwise.

### Soil and Growing Conditions

Soil composition was 10% silica sand, 42% sphagnum moss peat (Presto Durpes UAB, Vilnius, Lithuania) and 48% black peat and wood fiber (Ecomix, Vialca S.R.L., Uzzano, Italy). The soil was homogenized, passed through a 5 mm sieve, sterilized by baking at 120°C for 1 h, then held at 50°C for around 72 h until a constant weight had been attained. Seedlings were raised directly from uncoated seeds for 4 weeks in small pots under a 14 h photoperiod provided by 300 μmol m^-2^ s^-1^ metal halide lamps, with a 23/16°C day/night temperature and a constant relative humidity of 50%. They were then transplanted into 9 L pots and irrigated with 500 mL tap water (pH 7.5, EC 0.6–0.7 dS m^-1^) every 2 days. The soils’ EC and pH were monitored following the EPA method 9045D and 9050A.

Pots were fertilized weekly by adding 200 mL of 2% w/v blood meal (Guaber S.R.L., Bologna, Italy). Plants were raised in a greenhouse providing a day/night temperature of 25–30/13–16°C, with the natural light supplemented by 14 h per day of 300 μmol m^-2^ s^-1^ light provided by metal halide lamps. Soil, tap water and blood meal were sampled at the beginning and at the end of treatments.

### As and Si Treatments

Each cultivar, treatment type and treatment duration combination was represented by four plants. The treatments were initiated on plants grown for 100 days, coinciding with ripening of the first fruits. The three treatments were: nt (non-treated), As, and As+Si. For the As treatment, each pot was watered a single time with 2 L of 5 mg L^-1^ NaAsO_2_, for the As+Si treatment, with 2 L of 5 mg L^-1^ NaAsO_2_ combined with 2 mg L^-1^ CaSiO_3_. CaSiO_3_ is 10 mg 100 mL^-1^ water soluble at 20°C, at the concentration of 2 mg L^-1^ at room temperature the salt was completely solubilized. The nt plants received no supplementation. Samples were taken immediately before the treatment commenced (t0), after 48 h (t48h) and after 14 days (t14d). A visual assessment of the effects of As and As+Si on leaf health and number, flowering, fruit size and number was performed. The two cultivars compared were Aragon and Gladis. The whole root system was collected and also middle leaflets of the same age and positioned between the 6th and 8th nodes up the stalk. Fruits comparable in size and at the same developmental stage, according to ‘days after flower anthesis and color, were harvested (Klee and Giovannoni, 2011). All sampled fruits were positioned between the 6th and 8th leaf nodes. Fruits were washed in deionized water: the pericarp and cuticle were retained, while the placenta and seeds were discarded. According to the analysis to be performed, fruits were oven-dried, snap frozen in liquid nitrogen and stored at -80°C until use, or assayed immediately after harvest.

### Arsenic (As) Content Determination

Plant tissue As concentration was obtained using hydride generation atomic absorption spectrometry (HG-AAS). Following Marmiroli et al. (2014), root, leaf, fruit and soil samples were oven-dried, and ground to powder. A 300 mg (dry weight) aliquot of the powdered plant material was digested in 15 mL 14.6 M HNO3 for 60 min at 165°C. The resulting solution was subsequently diluted to 6.7 M HNO3 using distilled water. The soil samples were digested in 20 mL 14.6 M HNO3 / 10 mL 30% H2O2 for 60 min at 165°C, followed by 15 min at 230°C. The absorbance of each sample was read at 189 nm using a AA240FS instrument (Agilent Technologies, Santa Clara, CA, United States) equipped with vapor generator assembly (Varian VGA 77). The absorbances were converted into As concentrations via a standard curve based on a 10,000 ppm standard solution of high purity (>99%) As (Agilent Technologies, Santa Clara, CA, United States). All analyses were performed in triplicate. Arsenic concentration was measured in soil, tap water and blood meal following the same method.

### Silicon (Si) Content Determination

Inductively Coupled Plasma Optical Emission Spectrometry (ICP-OES) was employed to determine Si content of fruits, Si extraction followed van der Vorm (1987), with modifications. Briefly, plant material was dried and powdered, after which a 300 mg sample was reduced to ash in a muffle furnace for 3 h at 550°C. The ash was suspended in 12.5 mL 0.08M H_2_SO_4_ (Carlo Erba, Milan, Italy), added with 0.5 mL 23 M HF (Acros Organics, Geel, Belgium); the suspension was shaken for 1 h, then left overnight. The Si content of the resulting solution was measured by ICP-OES using an Optima 7300 DV device (Perkin Elmer, Waltham, MA, United States). All analyses were performed in triplicate. The instrument parameters were set as follows: power 1.4 kW; plasma gas flow rate 15 L min^-1^; nebulizer gas flow rate 0.78 L min^-1^; auxiliary gas flow rate 0.2 L min^-1^; sample flow rate 0.85 mL min^-1^; Si wavelengths 251.619 nm and 212.422 nm. A calibration curve was prepared from a Si standard solution (Perkin Elmer, Waltham, MA, United States), and used to convert the sample absorbances into Si concentrations.

### SEM/EDX Microanalysis

Portions of root, shoot and fruit washed deionized water, were cross-sectioned using carbon steel lancets (Incofar, Modena, Italy) and dried at room temperature. Microscope slides were prepared according to Marmiroli et al. (2014). Briefly, cross section of roots, shoots and fruit pericarp (with external cuticle) were positioned on carbon tape covered SEM stubs and coated with graphite. For each cross section, secondary electrons (SE) images, tissues distribution maps of As (Kα1) and Si (Kα1) emitted X-ray, and line-scan analysis, for comparing the relative elements abundance, of As, Si, Ca, K were evaluated using a scanning electron microscope (SEM) (Jeol 6400, Osaka, Japan) combined with an Oxford Si(Li) energy dispersive X-ray analyzer (EDX) operated by LINK ISIS software (Oxford Instruments, Oxford, United Kingdom). Operating parameters were: electron beam acceleration voltage: 20 keV, live time: 60 s, working distance: 11–13 mm, penetration depth for electron beam: 2–3 μm; acquisition times for maps and line-scans: between 4 and 6 h. All other parameters were set according to Marmiroli et al. (2014).

### H_2_O_2_ Content

H_2_O_2_ was quantified via a colorimetric method, following Junglee et al. (2014). Briefly, liquid nitrogen snap-frozen fruits were ground to powder of which a 100 mg sample was extracted in 1 mL of one part 0.1% (w/v) TCA (Honeywell Riedel-de Haen^®^, Seelze, Germany), one part 10 mM PBS (pH 7) and two parts 1M KI. The homogenate was centrifuged (12,000 *g*, 4°C, 15 min) and the supernatant held for 20 min at room temperature, after which the absorbance was read at 390 nm using a Varian Cary 50 spectrophotometer. The absorbances were converted into H_2_O_2_ concentrations via a standard curve based on a commercial H_2_O_2_ preparation (J.T. Baker, Deventer, Holland).

### Lipid Peroxidation

The extent of lipid peroxidation was assayed using the TBA test, which assays for the presence of MDA. Following Murshed et al. (2008), fruits were snap-frozen in liquid nitrogen, ground to a powder and a 200 mg sample was suspended in 1 mL 0.1% (w/v) TCA and centrifuged (12,000 *g*, 15 min). A 0.5 mL aliquot of the supernatant was added to 1 mL 0.5% (w/v) TBA in 20% (w/v) TCA and held at 95°C for 30 min, the reaction was then quenched by immersion in ice bath. After a brief vortex, supernatant’s absorbance was read at 532 nm using a Varian Cary 50 spectrophotometer. Absorbances were corrected by subtracting the reading made at 600 nm and converted into MDA contents via a standard curve based on a commercial preparation of MDA.

### Lycopene and Total Carotenoid Content

Following Fish et al. (2002), 1 g of fresh fruit was homogenized in a mortar in 5 mL distilled water and the resulting homogenate was kept on ice in the dark. Between 0.4 and 0.6 g of the puree was mixed with 5 mL 0.05% (v/v) 2,6-di(1,1-dimethylethyl)phenol] in 95% acetone, 5 mL 96% ethanol and 10 mL >99% hexane. Samples were kept on ice in the dark and shaken at 180 rpm for 15 min, after which 3 mL of deionized water were added and thoroughly mixed. The tubes were left at room temperature for 5 min to permit phase separation. The absorbance of the upper layer (hexane) was read at 503 nm (lycopene) and 450 nm (carotenoids) using a Varian Cary 50 spectrophotometer. Lyc content in mg per kg of tissue fw was given by A_503_^∗^ 31.2/g tissue. Carotenoids content was calculated from a calibration curve using β-carotene (type I, >93%) as the standard and was expressed as mg of β-carotene equivalents per kg of tissue fw.

### Ascorbate Redox State

The AsA and total ascorbate (ascorbate plus dehydroascorbate) contents were determined following Kampfenkel et al. (1995). Fruits were snap-frozen in liquid nitrogen and a 200 mg sample was ground in liquid nitrogen and suspended in 1 mL 6% (w/v) TCA. The homogenate was left on ice for 15 min, then centrifuged (16,000 *g*, 4°C, 10 min). A 200 μL aliquot of the supernatant was combined with 200 μL 10 mM DTT and 400 μL 0.2 mM PBS (pH 7.4). After a 15 min incubation at 42°C, the solution was made up to 0.5% w/v with NEM; after a further 1 min, 3 mL of a solution comprising five parts 10% (w/v) TCA, four parts 42% (v/v) H_3_PO_4_ (J. T. Baker, Center Valley, PA, United States), four parts 4% (w/v) 2,2-bipyridyl in 70% (v/v) ethanol, and two parts 3% (w/v) FeCl_3_. After vigorous mixing and incubation at 42°C for 40 min, the absorbance was read at 525 nm using a Varian Cary 50 spectrophotometer. The content of the reduced form of AsA was determined using the same protocol, with 0.2 mM PBS replacing DTT and NEM. A standard curve was constructed based on a commercial preparation of ascorbic acid. The AsA redox state was calculated from the expression (AsA)/(AsA + DHA)^∗^100.

### Total Phenolic Content

The total phenolic content was measured on methanol extracts of fruits prepared according to Capanoglu et al. (2008). Briefly, liquid nitrogen snap-frozen fruits were ground to a powder. A 100 mg aliquot combined with 1 mL 75% methanol was sonicated (Transsonic T460, Elma Schmidbauer GmbH, Singen, Germany) for 15 min at 35 kHz, centrifuged (1000 g, 4°C, 10 min), the pellet was subjected to a second round of extraction, two resulting supernatants were pooled and stored at -20°C until use.

Following Singleton and Rossi (1965), a 100 μL aliquot of the methanolic extract was mixed with 0.75 mL Folin-Ciocalteu reagent (a mixture of phosphomolybdate and phosphotungstate), allowed to stand at 22°C for 5 min, after which 0.75 mL 60 g L^-1^ sodium bicarbonate was added. After 90 min at 22°C, the absorbance was read at 725 nm using a Varian Cary 50 spectrophotometer. The total phenolic content was calculated from a calibration curve using GA as the standard. Total phenolic contents are given as μg GA equivalent per g tissue (fw).

### ABTS and DPPH Assays

The ABTS (2,2′-azino-bis (3-ethylbenzothiazoline-6-sulphonic acid) and DPPH (1,1-diphenyl-2-picryl-hydrazyl) assays were used to estimate the antioxidant activity of methanol extracts of fruits (as described in section *Total phenolic content*). Following Re et al. (1999), a 7 mM ABTS aqueous solution was oxidized by the addition of 2.45 mM potassium persulfate then incubated at 4°C for 16 h in the dark. The ABTS^•+^ radical solution obtained was held at 30°C, diluted in methanol, the absorbance was read at 734 nm (Varian Cary 50 spectrophotometer). After the addition of 1 mL ABTS^•+^ solution to a 10 μL aliquot of the above methanolic extract, the sample was held at 30°C for 5 min and the absorbances read at 734 nm after 5, 10, 15 and 20 min. Trolox (6-hydroxy-2,5,7,8-tetramethylchromane-2-carboxylic acid) was used to generate the standard curve. The radical inhibition percentage (I_ABTS_) was calculated from the expression ((A _ABTS_^•+^ - A _sample_)/ A _ABTS_^•+^) ^∗^ 100%. Following Brand-Williams et al. (1995), 1.95 mL of a freshly prepared solution of 0.06 mM DPPH in methanol was added to 50 μL of the methanolic extract, and held for 30 min at room temperature before the absorbance was read at 520 nm using a Varian Cary 50 spectrophotometer. The reading was repeated after 40 and 50 min of incubation to ensure that a steady state had been reached. Trolox was used to generate the standard curve. The radical inhibition percentage (I_DPPH_) was calculated from the expression (A _DPPH_ - A _sample_)/A _DPPH_) ^∗^ 100%.

### Glutathione Redox State

To estimate the glutathione (GSH) content, aqueous extracts were prepared following Nath et al. (2014). Snap-frozen tissues were homogenized in sterile distilled water (one part of fruit material to two parts of water) and the slurry was centrifuged (15,000 *g*, 4°C, 15 min). The supernatant was retained and stored at -20°C. The reduced GSH content was estimated following Griffith (1980) by the addition of 20 μL of the aqueous extract to 180 μL of potassium phosphate buffer (pH 7.5), 0.1 mM EDTA and 6 mM 5,5-dithiobis-(2-nitrobenzoic acid). After 10 min at 30°C, the absorbance was read at 412 nm using an iMark^TM^ microplate absorbance reader microplate reader (Bio-Rad). Total glutathione content (GSH+GSSG) was measured after reduction of GSSG to GSH by adding 2 mM NADPH and 1U glutathione reductase. GSH content was estimated using GSH as a standard and the glutathione redox state was calculated from the expression (GSH)/(GSH + GSSG)^∗^100%.

### Statistical Analysis

Statistical calculations were based on routines implemented either in IBM SPSS v. 23.0 (Chicago, IL, United States^3^) or in R v3.3.1.^4^ Further details are given in the Supplementary Materials. For all analyses a minimum of *n* = 4 biological replicates were utilized.

## Results

### The As and Si Content of the Tomato Plant

**FIGURE 1:**
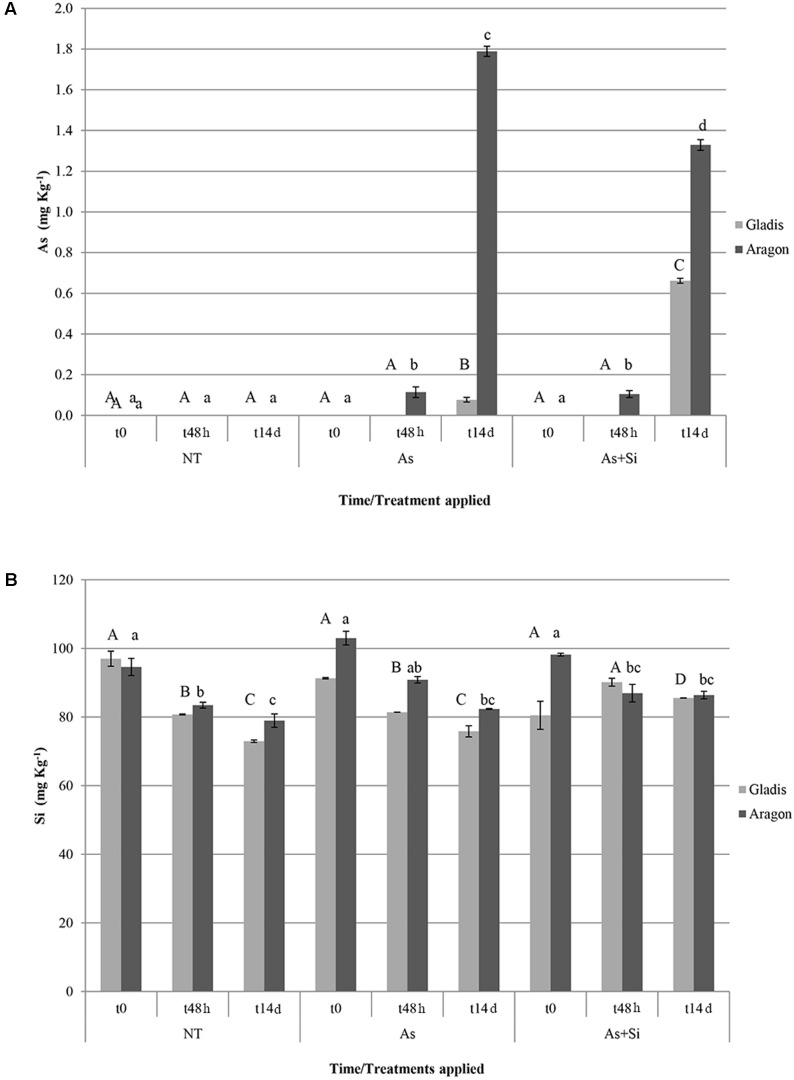
Arsenic **(A)** and silicon **(B)** concentration (in mg Kg^-1^ d.w.) in fruits of the cultivars Aragon and Gladis. Different superscript letters above histograms indicate significant differences according to ANOVA followed by *post hoc* Tukey’s HSD test for multiple comparisons analysis (*p* ≤ 0.01); upper case letters are for Gladis, lower case letters for Aragon. Values equal to 0 means below detection limit (BDL).

At t48h and t14d, the As content of fruit set by Aragon plants exposed to either the As or As+Si treatment was significantly higher than that of fruit set by the nt plants and by Gladis. At t14d, the As content of fruit set by Aragon plants was significantly lower under As+Si treatment in respect to As treatment. The As content in fruits of Gladis was higher under As+Si application than under As (**Figure 1A**). Overall, the fruit As content was at least twice as high in Aragon than in Gladis (**Figure 1A**). At t48h and t14d, in both cultivars, addition of Si reduced root uptake of As (less so in Aragon) but promoted its translocation to the aerial parts (**Figure 1A** and Supplementary Figures S1A,B). Arsenic content in soil, tap water and blood meal was under the detectable threshold.

**FIGURE 2:**
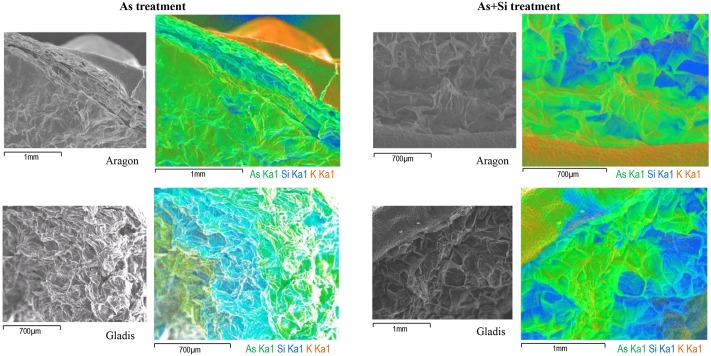
SEM/EDX fruit multi-element distribution maps. Fruit Secondary Electrons (SE) image, As (Kα1), Si (Kα1), K (Kα1) emission X-ray multi-element map, for Aragon **(Top)** and Gladis (bottom) under As **(Left)** and As+Si **(Right)** treatments. The map colors code is: green for As, blue for Si, and orange for K. In SE images black bar at bottom left indicates cross section size.

The present analysis suggests that the Si content of the fruit differed between the two cultivars (**Figure 1B**), and that it was influenced by both the nature of the treatment and the time of exposure; the interaction between these IVs was also significant (Supplementary Table S1). Fruit Si concentration decreased over time in both the As and the nt plants, but not in the As + Si treatment, in general, supplementation of As decreased Si allocated to the fruit of both cultivars (**Figure 1B**). At t14d, the Si concentration was consistently higher in the fruit set by Aragon than in that set by Gladis when the plants were exposed to either nt or As, but the cultivar difference disappeared in fruit set by plants exposed to As+Si (**Figure 1B** and **Table 1**). In fruits of Aragon, Si and As concentrations were positively correlated at t48h, negatively at t14d, in fruits set by Gladis the element concentrations were non-correlated at t48h, positively correlated at t14h (**Figures 1A,B**).

**Table 1:** Multivariate analysis of variance analysis of fruits from tomato cultivars Aragon and Gladis.

IV main effect and interactions	Test statistic	Exact value	*F*	H. df	E. df	Signif.	Effect size η*^2^ (%)*
Cultivar	Wilks’s Λ	0.007	265.981	11.000	21.000	^∗∗∗^	99.3
	Pillai’s V	0.993	265.981	11.000	21.000	^∗∗∗^	99.3
Treatment	Wilks’s Λ	0.001	62.080	22.000	42.000	^∗∗∗^	97.0
	Pillai’s V	1.924	50.668	22.000	44.000	^∗∗∗^	96.2
Time	Wilks’s Λ	0.000	253.134	22.000	42.000	^∗∗∗^	99.3
	Pillai’s V	1.983	236.519	22.000	44.000	^∗∗∗^	99.2
Cultivar ^∗^ Treatment	Wilks’s Λ	0.002	39.706	22.000	42.000	^∗∗∗^	95.4
	Pillai’s V	1.898	37.264	22.000	44.000	^∗∗∗^	94.9
Cultivar ^∗^ Time	Wilks’s Λ	0.003	34.482	22.000	42.000	^∗∗∗^	94.8
	Pillai’s V	1.465	5.477	22.000	44.000	^∗∗∗^	73.3
Treatment ^∗^ Time	Wilks’s Λ	0.000	40.881	44.000	82.295	^∗∗∗^	95.0
	Pillai’s V	3.662	23.666	44.000	96.000	^∗∗∗^	91.6
Cultivar ^∗^ Treatment ^∗^ Time	Wilks’s Λ	0.000	31.376	44.000	82.295	^∗∗∗^	93.6
	Pillai’s V	3.644	22.341	44.000	96.000	^∗∗∗^	91.1


*MANOVA assumptions were verified by testing for the homogeneity of covariances matrices (Box’s test was non-significant, *p* = 0.26) and a significant Bartlett sphericity test (*p* > 0.01). The multivariate statistics parameters considered, within a confidence interval of 99%, were Wilk’s Λ (lambda) and Pillai’s V. H.df: number of degrees of freedom for the hypothesis, E.df: number of degrees of freedom for the error term, Signif: significance value (^∗∗∗^*p* ≤ 0.001); η^*2*^ is the multivariate effect size (%).*

### Multivariate Analysis of Variance (MANOVA)

The full MANOVA on fruits is presented in **Table 1**. Both Wilk’s Λ and Pillai’s trace were highly significant (*p* < 0.001) for each of the single IVs (treatment, exposure time and cultivar), for the two-way interactions (treatment^∗^exposure time, treatment^∗^cultivar, exposure time^∗^cultivar) and the three-way interaction treatment^∗^exposure time^∗^cultivar. The univariate three-way ANOVAs revealed that the Lyc content did not vary significatively within each cultivar; that the glutathione redox state and the MDA content were not responsive to the treatment type; and that the ABTS assay outcome and the MDA content were only slightly affected by the exposure time (Supplementary Table S1). The non-significant variables within the cultivar^∗^treatment interaction were glutathione redox state and Lyc contents, within the cultivar^∗^exposure time interaction, the carotenoid and Si contents did not vary; within the treatment^∗^exposure time interaction, the total phenolic and MDA content did not change significatively, finally, in the three-way interaction the ABTS radical inhibition did not vary (Supplementary Table S1). HSD Tukey’s *post hoc* tests were performed for the IVs cultivar, exposure time, and treatment (**Figures 1A,B**).

### SEM/EDX Analysis of Elements Distribution

**FIGURE 3:**
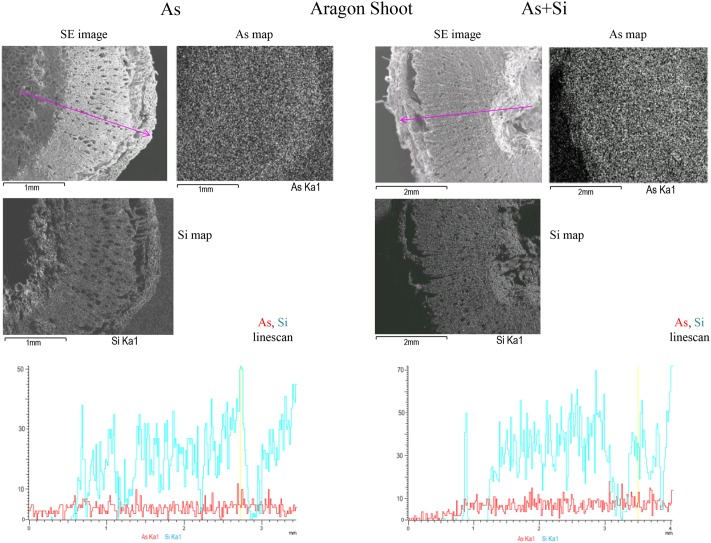
SEM/EDX multi-element distribution maps in Aragon shoots treated with As or As+Si. Shoot Secondary Electrons (SE) image, As (Kα1), Si (Kα1), emission X-ray element distribution maps and As and Si line-scan for relative concentrations for As treatment are on the **Left**, As+Si on the **Right**. The maps are in shades of gray: light gray is were elements are more concentrated, dark for lower concentrations. The pink arrow in the SE image indicates the line-scan transect, in line-scans red is for As, blue is for Si. In SE images black bar at bottom left indicates cross section size.

Only samples treated for 14 days showed concentrations of As above the EDX instrumental detection limit, thus results for t48h were not reported. Relative concentrations of elements in line-scans were expressed in counts per second (cps), comparable across all the analyzed samples. Relative concentrations of As and Si within tomato root, shoot, and fruit measured with microanalysis (EDX) were in accordance with the quantitative results obtained through chemical spectrometry (AAS and ICP-OES) (**Figure 3** and Supplementary Figures S1, S2, S3A,B).

Arsenic showed the highest counts in roots of cv. Gladis treated with As, in all other cases the linescans showed that the relative amounts of As were similar (**Figure 3** and Supplementary Figures S2, S3A,B). In shoots of both cvs. under As and As+Si treatments As counts, reported in the respective linescans, were comparable with that in roots, however As in shoots was highest in Aragon treated with As+Si. Distribution maps showed that As in roots was mostly localized in cortex and epidermis but reached also the vascular bundles. In shoots As was evenly distributed in all tissues from the epidermis to the pith (**Figure 3** and Supplementary Figure S3B). Linescans showed that Si in roots and shoots of cv. Gladis in condition of As+Si treatment was more abundant than in roots and shoots of Aragon treated in the same way. Linescans evidenced also that Si was always in higher quantities than As in roots and shoots of all plants treated with As alone. Distribution maps showed that Si distribution pattern in roots and shoots was similar to As, although more concentrated in epidermis and in cortex rather than in vascular bundles, particularly in roots (**Figure 3** and Supplementary Figures S2, S3A,B). From linescans it appeared that the highest amount of As was found in fruits set by cv. Aragon treated with As and in those set by cv. Gladis treated with As+Si (**Figure 2** and Supplementary Figures S4A,B). There were no appreciable differences in Si counts in fruit of cv. Aragon treated with As and As+Si, while in Gladis the As+Si treatment increased Si counts as compared to the As treatment (Supplementary Figures S4A,B). The multi-element distribution maps showed that in fruits of both cvs. treated with As and As+Si, As and Si allocated mainly in the pericarp, Si was also found in the cuticle (**Figure 2** and Supplementary Figures S4A,B). We followed also the macronutrients, K and Ca which are of particular relevance because of their key role in tomato fruit biotic and abiotic stress resistance (Cuartero and Fernández-Muñoz, 1999; Dumas et al., 2003) and Ca can influence Si and As uptake and translocation (Islam et al., 2015; Pontigo et al., 2015). In roots and shoots of both cvs. treated with As and As+Si, K and Ca reached similar amounts in the internal tissues (pith, vascular bundles), with Ca higher than K in the roots and shoots cortex and epidermis (Supplementary Figures S5, S6, S8, S9). Conversely, in fruit of both cvs. K was more abundant than Ca, especially, within fruit of cv. Aragon (Supplementary Figures S7, S10).

### H_2_O_2_ Production and MDA Content

**FIGURE 4:**
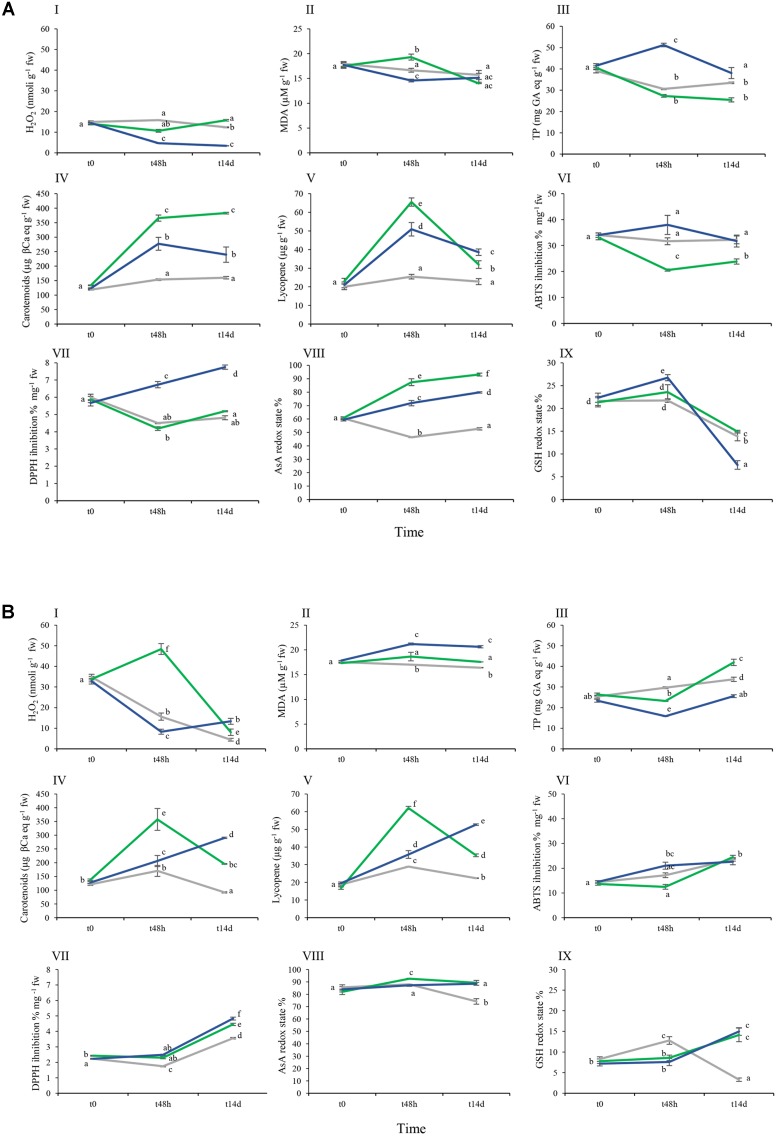
Effect of As and As+Si biochemical parameters in cvs. Aragon **(A)** and Gladis **(B)**. Lines of different colors are for different treatments: *gray*: nt, *green*: As, *blue*: As+Si. Black vertical bars indicate standard errors (se). Boxes from **I–IX**: H_2_O_2_, MDA, TPs, Carotenoids, Lycopene, ABTS, DPPH, AsA redox state, GSH redox state. Different superscript letters above the standard error (SE) bars indicate significant differences according to ANOVA followed by *post hoc* Tukey’s HSD test for multiple comparisons analysis (*p* ≤ 0.01). Values equal to 0 means below detection limit (BDL).

H_2_O_2_ is a well-recognized agent of oxidative damage able to disrupt metabolic function and compromise cellular integrity (Halliwell, 2006). In fruit set by non-treated Aragon plants, the level of H_2_O_2_ fell only slightly between t0 and t14d, while in treated plants (both the As and especially the As+Si treatments), a much larger decrease was noted by t48h (**Figures 4A-I**). By t14d, the levels in the fruit set in the nt fruit and in the As treatment were comparable. In contrast, fruit set by non-treated Gladis plants experienced a gradual fall in H_2_O_2_ content. The levels differed significantly between the treatments at t48h: a substantial increase occurred in the As treatment samples and a decrease in the As+Si treatment ones (**Figure 4B-I**).

Malondialdehyde is recognized as a biomarker for lipid peroxidation. The MDA content of Gladis fruit was unaffected by the As treatment, but when assayed at t48h had increased in the As+Si treatment and was still high at t14d (**Figure 4B-II**). Nevertheless, the implication from the MANOVA was that for both cultivars the MDA content did not significantly depend on the type of treatment, and was only slightly dependent on the exposure time (**Figures 4A-II,B-II** and Supplementary Table S1). Both H_2_O_2_ and MDA concentrations in fruits were highly dependent on the cultivar (**Figures 4A-I,II,B-I,II** and Supplementary Table S1).

### The Content of Phenolics

Phenolic compounds (phenylpropanoids) have considerable physiological and morphological importance for plant growth, reproduction and the response to biotic and abiotic stress (Winkel-Shirley, 2002). The total phenolic content (TP) of tomato fruit is highly cultivar dependent (Luthria et al., 2006). Here, Aragon fruit set by non-treated plants contained at t0 around 40 μg on a fw basis, and those of Gladis around 25 μg GA eq g^-1^ (**Figures 4A-III,B-III**). In fruits of nt Gladis, TP content increased over time; in nt Aragon, the content fell over time (**Figures 4A-III,B-III**). After 48 h exposure to the As treatment, TP content of Aragon fruit was lower than that of the comparable nt fruit. However, for the As+Si treatment group the effect was an increase of TP at 48 h, which after 14 days declined to a concentration similar to that found at the start of treatment (**Figure 4A-III**). Fruit of Gladis plants exposed to the As treatment showed a significant increase in TP after 14 days, while the As+Si treatment resulted in a significant decrease of the TP after 48 h, followed by a recovery to the initial value (**Figure 4B-III**). According to the three-way ANOVA, the type of treatment accounted for only a limited portion of the TP variability, which is largely explained by the time of treatment and the interaction among all three IVs (Supplementary Table S1).

### Carotenoid and Lycopene Content

The major carotenoids present in the tomato fruit are Lyc and β-carotene (Agarwal and Rao, 2000). Abushita et al. (2000) have demonstrated that the carotenoid content of the fruit can vary from 68 and 125 μg g^-1^ fw between cultivars, while that of Lyc ranges from 77 to 116 μg g^-1^ fw. In Aragon, the fruit carotenoid and lycopene contents remained steady in the non-treated plants, but rose at t48h in response to both the As and (strongly) the As+Si treatment. At t14d, for both treatments, the level of carotenoids was the same as at t48h as, but Lyc was significantly reduced (**Figures 4A-IV,V**). At t14d in Aragon fruits lower levels of Lyc were measured under As treatment than under As+Si (**Figure 4A-V**). In Gladis fruit, carotenoid content in the nt plants rose sharply at t48h and then dropped significantly at t14d. Under As treatment, fruit carotenoid content assayed at t48h was much higher than at t14d, while with As+Si treatment, fruit carotenoid content rose first at t48h and then again at t14d (**Figure 4B-IV**). The Lyc content in Gladis fruit followed the time trend shown by carotenoids. The three-way ANOVA, however, showed that Lyc was not affected by genotype, but by time and type of treatment and their interactions (Supplementary Table S1).

### ABTS and DPPH Assays

The assays record the total capacity of cells to reduce two synthetic radicals (ABTS^+^ radical cation and DPPH neutral radical); however, the extraction method for fruit tissue metabolites might cause partial loss of photosensitive molecules such as tocopherols. In the fruit of non-treated (nt) Aragon plants, the presence of ABTS targets did not change over time (**Figure 4A-VI**). When challenged by As, the scavenging activity toward ABTS radicals decreased at t48h then rose at t14d (**Figure 4A-VII**). Supplementation with Si had the opposite effect on the assay, whose activity rose then fell, despite the higher standard deviation (**Figure 4A-VI**). In the case of the DPPH assay, there was no discernible response to the As treatment compared to nt, but for the fruit of plants exposed to As+Si, there was a positive response over time (**Figure 4A-VII**). In Gladis fruit there was a higher presence of ABTS targets by t14d than at t0 in each of the treatments (**Figure 4B-VI**) According to the Three-way ANOVA, the ABTS assay was less affected by exposure time than was DPPH (Supplementary Table S1).

### AsA and DH-AsA Content

Ascorbate is the most abundant antioxidant present in plant cells; it is involved in the removal of H_2_O_2_ via the AsA-glutathione cycle and is a key player in the AsA-glutathione pathway, responsible for H_2_O_2_ and ROS metabolism in plants (Singh et al., 2006; Foyer and Noctor, 2011). The AsA content of the tomato fruit during its ripening is known to be cultivar-dependent (Mellidou et al., 2012).

For nt plants of both cultivars, fruit AsA redox state remained steady during the experiment, at about 60% in Aragon and 80% in Gladis (**Figures 4A-VIII,B-VIII**). In Aragon, the treatments were associated with a significant increase in the AsA redox state over time; this was particularly the case in the As treatment (**Figure 4A-VIII**). In Gladis the AsA redox state was less sensitive to both treatment type and exposure time than in Aragon. The results evidenced a cultivar dependency of AsA/DHA redox state influenced by the exposure time and the type of treatment, as revealed by the three-way ANOVA and follow-up *post hoc* tests (**Figures 4A-VIII,B-VIII** and Supplementary Table S1).

### Glutathione Redox State

Plants and animals rely on redox activity of the low molecular weight thiol glutathione. Under non-stressed conditions, the cellular glutathione pool is primarily in its reduced state (GSH), maintaining a GSH:GSSG ratio of at least 20:1 (e.g., Mhamdi et al., 2010).

The glutathione redox state in Aragon fruit at t48h under both the As and As+Si treatments was different to that of the non-treated (nt) plants. After 14 days a significant decrease of the GSH redox state was found in treated and nt samples (**Figure 4A-IX**). GSH/GSSG in Gladis at t48h under As and As+Si was different from the nt group. At t14d a significant increase in GSH/GSSG was observed in treated samples (**Figure 4B-IX**). In the fruit of non-treated plants of both cultivars, the glutathione redox state showed a significant decrease only by t14d. The Three-way ANOVA did not suggest any significant change in the glutathione redox state due to the treatments, but an effect of both exposure time and cultivar was identified (Supplementary Table S1).

### Dimension Reduction and Heat-Maps

**FIGURE 5:**
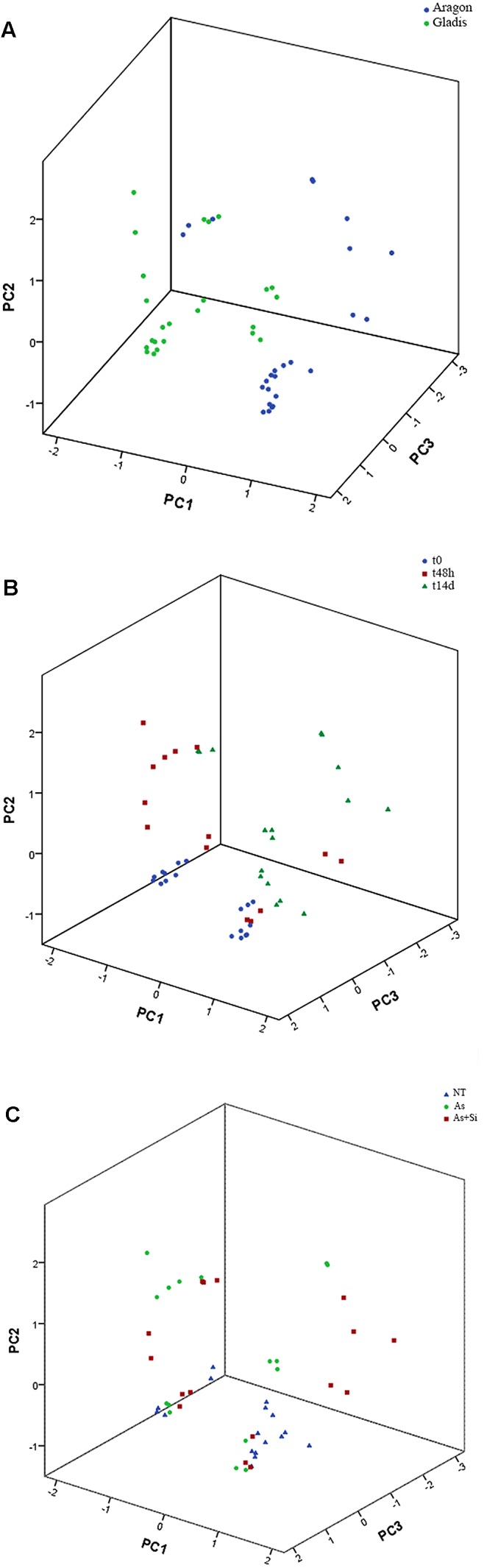
Principal component analysis of the fruits, considering the three variables cultivar **(A)**, treatment **(B)** and time of treatment **(C)**. KMO (Kaiser–Mayer–Olkin) index = 0.67. The components extraction criteria were: eigenvalue λ > 1 and varimax (orthogonal) rotation. The total proportion of the overall variance explained (e.v.) was 74.7%. PC1: λ = 3.9, e.v. = 36.0%. PC2: λ = 2.6, e.v. = 23.7%. PC3: λ = 1.7, e.v. = 15.0%.

**FIGURE 6:**
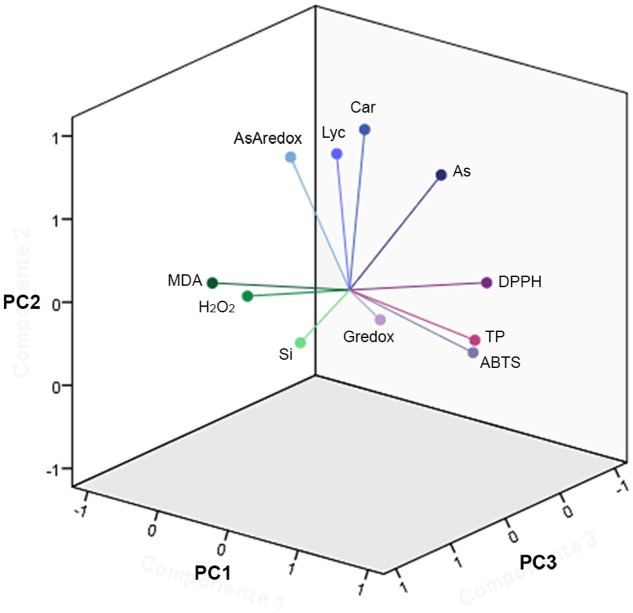
Principal component analysis vectors. Three-dimension (3D) representation of the 11 vectors for the PCA of the fruit for both cultivars, as in **Figure 5**, utilizing the loading values in Supplementary Table S2. The component extraction criteria were: eigenvalue λ > 1 and varimax (orthogonal) rotation. The total proportion of the overall variance explained (e.v.) was 74.7%. PC1: λ = 3.9, e.v. = 36.0%. PC2: λ = 2.6, e.v. = 23.7%. PC3: λ = 1.7, e.v. = 15.0%. Vectors represent the amount of total variance, for all treatment types and treatment times, apportioned in each parameter.

**FIGURE 7:**
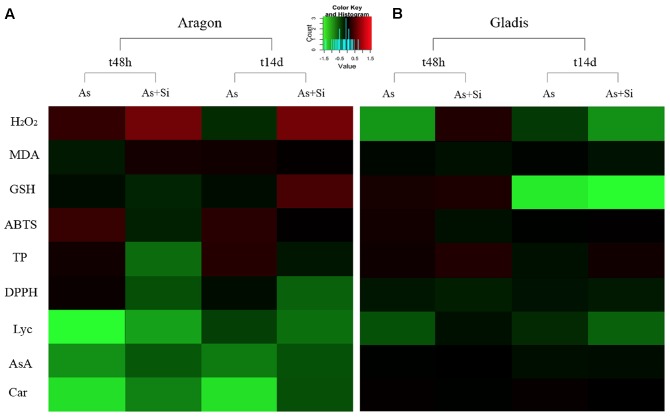
Heat-maps for cv. Aragon **(A)** and cv. Gladis **(B)**, of each parameter listed on the left. Parameters values were obtained by subtracting from the control (nt) the respective value of the treatment (As or As+Si) either after short-term (t48h) or long-term (t14d) exposure, then normalizing by the nt value. *Green*: control lower than treatment, *red*: control higher than treatment, *black*: equal values.

Dimension reduction provides a means to recognize patterns in multivariate data sets by constructing weighted combinations of the variables which are able to explain a major portion of the variance (Henson and Roberts, 2006; De Ron et al., 2016; Popović et al., 2016; Ranjit et al., 2016; Afonso et al., 2017). Three components/factors were extracted using the Kaiser criterion [eigenvalue (λ) > 1] for the dependent variables set for the fruits (**Figures 5**, **6**, Supplementary Figure S11, and Table S2). FA factors explained 66.3% of the common variance; PCA components explained 74.7% of the total variance (Supplementary Table S2). The loading coefficients for the components and factors were similar, therefore PCA and FA representations in 3-D space showed only minor differences (**Figure 6** and Supplementary Table S2). Dependent variables G redox, ABTS, DPPH, and TP all loaded mainly on component/factor 1; AsA, Lyc, carotenoids, and As on component/factor 2; MDA, H_2_O_2_, and Si loaded on components/factors 3. **Figure 6** represents dependent variables vectors in 3-D space. The most striking result was the sharp difference between the two cultivars (**Figure 5A** and Supplementary Figure S11). PCA plot groupings according to time or type of treatment (**Figures 5B,C** and Supplementary Figure S11) showed that both time (t0, t48h, t14d) and type (nt, As, As+Si) of treatment influenced the spatial distribution of the experimental points in equal measure, but still less than the cultivar type. Within each cultivar, the reprogramming effect of treatment on the metabolic and physiologic parameters were pictured using intensity heat-maps (**Figures 7A,B**). The differences between control (nt) and treatments (As or As+Si) for each parameter were calculated within short-term (t48h) or long-term (t14d) exposure. Green shades showed how parameters’ values increased in treated plants in respect to non-treated, red shades for the reverse. In both cvs. As exerted a larger molecular reprogramming at t48h than at t14d, while Si was less effective in restoring altered parameters to their nt levels in cv. Aragon than in cv. Gladis. In both cvs., H_2_O_2_, GSH, TP and ABTS were highly responsive to treatments at both t48h and t14d, the other parameters were more affected at t48h than at t14d (**Figures 7A,B**).

## Discussion

Excessive As in plants is thought to induce oxidative stress, and thus enhance the production of ROS (Sharma, 2012). More recently, it has been shown that it can also negatively impact photosynthesis, cellular electrolytes, and nutrients status (Finnegan and Chen, 2012; Sanglard et al., 2014). Oxidative stress responses vary according to a cell’s physiological state (Vanden Driessche et al., 2000) and the maturation process in a climacteric tomato fruit is a case in point (Klee and Giovannoni, 2011). Development of the tomato fruit, after ethylene peak and respiratory burst, proceeds through several major stages characterized by rapid cell division, expansion and endo-reduplication to increase fruit weight (Saltveit, 2005). During true ripening, extensive metabolic reorganization takes place (Giovannoni, 2004), marked by the conversion of chloroplasts into chromoplasts, carotenoid accumulation and chlorophyll degradation (Pesaresi et al., 2014; Suzuki et al., 2015). Since ROS produced during ripening play a major role in aging and cell death (Jacobson, 1996), their level is tightly regulated by antioxidants such as carotenoids and ROS-scavenging enzymes (Kumar et al., 2016).

Thus ranges of antioxidant metabolites synthesized by plants to avoid damage induced by ROS as during an oxidative stress (Foyer and Noctor, 2011) are constitutively present in fruits, at different levels according to their ripening stage (George et al., 2004). Therefore the treatment with As of tomato plants, and the fact that substantial amounts of As were translocated into fruits, offered the opportunity of studying antioxidant metabolites synthesis when an exogenous stress (As) overlays the condition of endogenous metabolic rearrangement (ripening).

In the experiments reported, As concentration administered to plants was within the range used for published experiments on tomato and other plant species (Srivastava et al., 2007; Madeira et al., 2012). Arsenic concentrations measured in fruits were consistent with the literature (Madeira et al., 2012). There was a wide difference between the cultivars in the rate of As and Si uptake and translocation to shoots and fruits, which varied in time and concentrations (**Figures 1A,B** and Supplementary Figures S1A,B).

It was observed that under normal soil Si content (control), As positively hindered Si transfer to fruits, albeit in a cv-dependent way. Consistently, previous studies evidenced that the ability of tomato to take up and translocate As to fruits was strongly affected by genotype (Madeira et al., 2012).

Si accumulation is a feature of primitive land plants along with angiosperm species in the *Poaceae*, *Cyperacea*e and *Commelinaceae* families (Ma and Takahashi, 2002). A wide survey of the extent of Si accumulation by shoots in angiosperm species concluded that tomato has a very low uptake, averaging only around 1.5 ppm on a dry weight basis (Hodson et al., 2005). Recent studies have suggested that tomato organs vary significantly with respect to their Si accumulation in response to specific treatments, and unambiguous evidence has been presented for the existence of genetic variation for the trait (Cao et al., 2015; Kleiber et al., 2015). Supplementation with Si exerts a protective effect against many types of stress including As (Tripathi et al., 2013; Adrees et al., 2015; Sanglard et al., 2016).

In the present study a picture emerged in which the plant responses to As and As+Si are consequences of a complex network of effects and interactions. MANOVA data analysis emphasized the importance of capturing the response with a range of parameters and of employing two different genotypes and more than a single exposure time. The Si concentration administered here was within the range presented in the literature, especially for tomato plants (Gunes et al., 2007; Kleiber et al., 2015). Moreover, when supplementing the soil with CaSiO_3_ we added a concentration of Ca of 690 mg L^-1^for a total of 1.38 g per pot of 9 L each. This amount of Ca is remarkably lower than the basal Ca concentration in soils which could be between 0.6% and >10% (Havlin et al., 2013).

Most of the studies on As and Si uptake and transport have been performed in rice and Arabidopsis, where specific genes code for influx and efflux Si channels (OsLsi1, OsLsi2, AtNIP5;1) exploited also by As (Ma et al., 2008; Zhao et al., 2009). Unfortunately, to date, in the tomato genome no orthologous sequence of these or other As/Si specific transporters has been cloned (Norton et al., 2010; Pontigo et al., 2015). Therefore, data on As and Si distribution in tomato organs and tissues remains difficult to interpret in a mechanistic way.

EDX distribution maps evidenced how at t14d, in plants of both cvs. under As and As+Si treatment, As was taken up by roots, moved into the vascular bundles, transported to the shoots where through the xylem was translocated mainly to the fruit pericarp (**Figures 2**, **3** and Supplementary Figures S2, S3A,B S4A,B). Notably, under As and As+Si treatments, Si followed the same route as As for uptake, translocation to shoot and fruit, and accumulation in roots and shoots external tissues (**Figure 3** and Supplementary Figures S2, S3A,B). The variation in relative concentrations suggested that in both cvs. As and Si competed for transport and allocation mechanisms, although the difference in As and Si abundance between cvs. pointed to an overlapping with cultivar-related effects. Analogous cultivar-dependence was observed for Ca and K abundance within plants organs (Supplementary Figures S5–S10), in particular in fruit of cv. Aragon under both treatments Ca and K were sensibly higher than in fruit borne by cv. Gladis (**Figures 2**). It appeared that As and Si eventual allocation to fruit could dependent also on Ca and K homeostasis, in agreement with the key role played by both macronutrients in influencing biotic and abiotic stress response in tomato (Dumas et al., 2003; Islam et al., 2015). In general high concentrations of K in tomato fruit promote carotenoids biosynthesis therefore increasing the pool of anti-ROS molecules available to counter As oxidizing activity (Dumas et al., 2003). While in roots and shoots high levels of Ca are necessary to support the cell wall structure, in tomato fruit due to low transpiration and hydraulic isolation, Ca reaches concentrations around 2 mM (Bar-Tal et al., 2017), differently from other fruits (Qin et al., 2009), which might be consistent with a stress signaling role, as reported in *Arabidopsis* by Guo et al., 2016. Calcium triggers a stress signaling molecular cascade through the expression of several protein kinases (CDPK genes, CBL-interacting protein kinase (CIPK), calcineurinB-like protein (CBL) through Calmodulin binding (Das and Pandey, 2010; Truong and Carroll, 2013). Consistently, in a parallel study on proteome modulation in the same plants used in this study, we found for both cultivars a number of proteins involved in Ca signal transduction, such as Calmodulin, CBL-interacting protein kinase, and Calcium–dependent protein Kinase (Marmiroli et al., 2017).

H_2_O_2_ is a non-radical (molecular) ROS, while superoxide radicals ($O_{2}^{•-}$) and hydroxyl radical (OH^-^) are free radical ROS (Halliwell, 2006). A rise in H_2_O_2_ content is a typical plant response to As exposure (Islam et al., 2015) and is characteristic of the ripening fruit (Jimenez et al., 2002). This effect, which is generally mitigated by supplementation with Si (Pandey et al., 2016), was observed in fruits of both cultivars. However, H_2_O_2_ increase caused by As treatment was strongly time- and cultivar-dependent and moderately correlated with As levels within fruits (**Figures 1A**, **4A-I,B-I**, **7A,B**).

According to Jimenez et al. (2002), the MDA content of tomato fruit rises as it ripens, whereas in our case it rose only slightly with time. The difference could be due to the increased contents of phenolics and carotenoids that we observed, which are able to neutralize peroxide radicals and thereby control lipid peroxidation as well (Martínez-Valverde et al., 2002). Similarly to H_2_O_2_ levels, lipid peroxidation and As concentrations in fruits were only weakly related (**Figures 1A**, **4A-II,B-II**). During fruit ripening cell membrane were oxidized by As-induced and ripening-induced ROS increasing MDA fruit concentrations (Mondal et al., 2004; Verbrugger et al., 2009). Silicon restoring effect of the cell redox imbalance and reduction of MDA appeared to be effective only in the long term. Arsenic affects negatively also mitochondria outer and inner membranes and causes a further damage to mitochondrial functions by substituting Pi thus leading to respiration impairment (Finnegan and Chen, 2012). In our proteomic study of these same tomato fruit under As treatment, we found two reprogrammed proteins related to mitochondria: probable lipid-A-disaccharide synthase, mitochondrial (predicted); pentatricopeptide repeat-containing protein At1g80270, mitochondrial (predicted) involved in signal transduction, lipid biosynthesis and biogenesis/function respectively (Marmiroli et al., 2017). It is therefore likely that in our case As damaged both mitochondria structural membranes and function.

Phenolic compounds have considerable importance in plant growth and stress defense, in particular, thanks to their redox potential, they constitute powerful antioxidants (Winkel-Shirley, 2002; Taylor and Grotewold, 2005). In general, the TP content tends to be higher in the fruit than elsewhere in the plant, because antioxidant activity is specifically required to neutralize ripening-generated ROS (Klee and Giovannoni, 2011; Tohge et al., 2014). Therefore Si, which exerts a direct influence over phenylpropanoid synthesis (Dragišić Maksimović et al., 2007) here improved indirectly the ability of cells to quench As-induced free radicals by stimulating biosynthesis of anti-ROS metabolites (Savvas and Ntatsi, 2015). However, the total phenolic (TP) content of tomato fruits is highly cultivar dependent (Chandra et al., 2012). As a fact, this mechanism was stronger in fruit from Aragon than in fruit borne by Gladis (**Figures 4A-III,B-III**).

Color change in ripening tomato is due to chlorophyll degradation and carotenoid biosynthesis as described by Fraser et al. (1994). Lyc and β-carotene are the major carotenoids present in tomato. Lyc, which accounts for the redness of the fruits, is more effective in scavenging ROS than is β-carotene (Agarwal and Rao, 2000).

The carotenoid contents increase in response to the presence of As because of their role in detoxifying ROS (Chandra et al., 2012). As observed for phenylpropanoids, this process was strongly cultivar-dependent (**Figures 4A-IV,V,B-IV,V**). Carotenoids are synthesized in the chromoplasts, therefore these organelles, which substitute the chloroplast in ripening fruit, appeared to play a major role in ROS detoxification, in particular in alleviating stress symptoms induced by As treatment.

In fruits set by both cultivars the radical scavenging capacity toward ABTS^+^ and DPPH on the short term (t48h) was higher under As+Si treatment than under As treatment (**Figures 4A-VI,VII,B-VI,VII**) in accordance with the ability of Si to stimulate synthesis of ROS-quenching molecules (Panda et al., 2010; Pontigo et al., 2015). The capacities measured in fruits to inhibit ABTS^+^ and DPPH were dependent on the type of treatment but mainly on the cultivar, probably because ripe fruits set by different cultivars display different antioxidant pools (George et al., 2004; Sánchez-Rodríguez et al., 2010). ABTS and DPPH assays mirrored more the time and treatment trends showed by TP rather than the trends displayed by carotenoids. PCA, FA, and heat-maps confirmed that ABTS and DPPH assays detected mostly the antioxidant mechanisms exerted by phenolics (**Figures 5**–**7**).

Ascorbate is involved in the removal of H_2_O_2_ via the AsA-glutathione cycle and is a key player in the AsA-glutathione pathway, responsible for H_2_O_2_ and ROS metabolism in plants (Singh et al., 2006; Foyer and Noctor, 2011). The recycling of AsA to DHA takes place in a glutathione-dependent reaction catalyzed by DHAR (Foyer and Noctor, 2011). A major problem in interpreting changes in the AsA/DHA ratio is that much of the DHA pool is spatially separated from the AsA pool. The latter accumulates in apoplasts, considered to be the site of its degradation, while the former is found within the cell where it acts as cofactor for several enzymes and is required for the synthesis of anthocyanins, flavonoids and glucosinolates (Turnbull et al., 2004). Ascorbic acid may participate in chloroplast-to-nucleus retrograde signaling (from the chloroplasts to the nucleus), which is of particular importance for the correct assembly of functional chloroplasts and the chloroplast-to-chromoplast transition characteristic of fruit ripening (Koussevitzky et al., 2007). The high AsA/DHA ratio observed at t48h and at t14d in nt fruits set by both cultivars reflected the need during ripening for a high apoplastic DHA concentration, driven by cell multiplication, enlargement and remodeling (**Figures 4A-VIII,B-VIII**). Under As treatment, fruit AsA/DHA ratios consistently increased to meet the demand for AsA both as a catalyst in ROS metabolism and as an As-induced ROS scavenger. Si was more effective at increasing the AsA/DHA ratio in fruit borne by Aragon than by Gladis at all times. A possible reason for the observed difference could be that AsA/DHA ratios in fruits of Gladis were already greatly increased by the oxidative stress caused by ripening. Concurrently, As treatment at t48h caused in fruit borne by Gladis a burst in H_2_O_2_ concentration likely to require an increase in the pool of AsA (**Figures 4B-I,VIII**).

Glutathione reacts with both ROS and DHA, which generates a close relationship between the H_2_O_2_ content of a cell and its glutathione status (Mhamdi et al., 2010). GSH and AsA form part of a networked antioxidative system in plants although they each are associated with a specific set of functions (Foyer and Noctor, 2011). Glutathione plays a leading role in plant response to As exposure, because both arsenate and arsenite have a high affinity for thiols; arsenate reduction is coupled to NADPH oxidation via the reduction of GSSG with the resulting glutathione serving as an electron donor for arsenate reductase (Ellis et al., 2006). At the same time, GSH acts as a component of ROS (and arsenite) detoxification and sequestration through phytochelatins (Ali et al., 2009; Foyer and Noctor, 2011). The glutathione redox state may be modified by Si because the element can influence membrane integrity, modulating electron leakage in the cell (Cao et al., 2015). The expected production of ROS associated with fruit ripening and As treatment favored GSSG over GSH (**Figures 4A-IX,B-IX**). However, supplementation with Si halved the basal GSH/GSSG ratio in Aragon, but doubled it in Gladis. Mellidou et al. (2012) have described a similar cultivar-dependence for GSH content during tomato fruit ripening. The presence of As, whether or not Si supplementation was provided, would have promoted the recruitment of GSH in order to chelate the As; the GSSG generated as a result would be available to restore AsA from DHA. As ripening progressed, there would have been a steady increase in the extent to which the glutathione pool existed in its oxidized state within the fruit. This process appeared to be cultivar dependent, as observed for AsA, since in fruit borne by Gladis the GSH content was lower than in fruit set by Aragon under As treatment, whereas the opposite happened when adding As+Si. However, on longer term (t14d) the GSH/AsA cycle, with or without Si addition, did not counter As allocation into fruits of both cultivars.

In this work we used dimension reduction multivariate statistics (Factor Analysis and Principal Component Analysis) to understand the interconnections among the chemical and biochemical variables measured in this study. Dimension reduction statistics searches for the smaller set of latent mathematical factors/component to represent the larger set of measured variables. FA explicitly focuses on the common variance among the variables which are linear functions of the (common) factors, plus an error term while PCA focusses on the total variance of all the variables, which are represented as linear functions of the components (Joliffe and Morgan, 1992). FA is also termed the “Common Factor” model whose goal is to understand the biological correlations among measured variables (Williams et al., 2012).

On the basis of the FA and PCA, whose factor loadings are reported in Supplementary Table S2 and visualized in **Figure 6**, we could infer that component #1 reflected the TP content, GSH redox state, ABTS and DPPH; component #2 represented mainly carotenoid and Lyc content, As concentrations and AsA redox state; while component #3 represented mainly the content of MDA, H_2_O_2_, and Si (**Figure 6** and Supplementary Table S2). A possible biological interpretation emerging from this approach was that factor #1 represents the bulk of the radical scavenging capacity, the primary ROS and part of the cell redox state; factor #2, isoprenoid metabolism, and hence the ripening process and As contamination; while factor #3 encompassed the effects of Si and its importance for part cell redox state. Notably, the two main hubs of ROS metabolism, AsA and GSH (Foyer and Noctor, 2011) are loaded separately orthogonal components We could infer from the spatial distribution of the experimental points according to PCA that, within each cultivar, both time and type of treatment influenced the variance of the dependent variables (**Figures 5B,C** and Supplementary Figure S11). This observation is in keeping with the MANOVA results (**Table 1** and Supplementary Table S1). One possible biological interpretation is that Arsenic toxicity, mainly in the form of oxidative stress, determined a chain of physiological and biochemical changes within ripening tomato fruit to produce a network of oxidizing/reducing mechanisms more complex than that observed in roots and shoots (Tripathi et al., 2015; Pandey et al., 2016). In this context the alleviation of As-induced stress effected by Si was less clear cut than in roots and shoots (Romero-Aranda et al., 2006; Gunes et al., 2007; Cao et al., 2015) and more effective on the long term. Heat-maps (**Figures 7A,B**) captured three molecular and physiological parameters highly responsive in the two cvs. at both short-term (t48h) and long-term (t14d) treatments, H_2_O_2_, GSH, TP, which are directly involved in oxidative burst and ROS scavenging (Foyer and Noctor, 2011).

This study proved that the damaging As and the protective Si reached tomato fruits during ripening and influence their content of antioxidant metabolites.

Here, two cultivars, both intended for industrial processing, were compared because they showed different fruit morphology and remarkable differences in response to As+Si at the level of the whole plant (Marmiroli et al., 2014). However, the genetic distance between the two cultivars was so small to be discriminated only by a powerful set of microsatellites (SSRs) (Marmiroli et al., 2014). This same genetic closeness between tomato cultivars intended for industrial processing was also found by Bauchet and Causse (2010) and Bolger et al. (2014). Moreover, responses to As and Si are determined by complex and polygenic traits (Norton et al., 2010) while total phenotypic variance rests not only on the very limited genetic distance between cultivars but also on the interaction between genotype and environment, which was high under the applied treatments (As and As+Si).

Reprogrammed synthesis of some essential metabolites and readjustment of ROS balance within the fruit constituted the main response observed here to As and As+Si treatment. Arsenic stress response in plants combines general stress features with specific genetic and molecular mechanisms ascribable to oxidative stress response (Panda et al., 2010; Finnegan and Chen, 2012). Expression of specific stress-response genes was reported in tomato fruit when plants were treated with As (Goupil et al., 2009). A detailed proteomic analysis on tomato fruit treated with As and As+Si showed a reprogramming of proteins from the abiotic stress family (Hsps, calmodulins, growth regulating factors, proteins ubiquitination), but also specific to oxidative stress response (sulfotrasferase, lipid metabolism proteins, and secondary metabolites biosynthetic enzymes) (Marmiroli et al., 2017). In conclusion, within fruit distinctive molecular background, AsA and GSH redox state, total carotenoids, TP, and H_2_O_2_ levels were optimal parameters to be considered as effective “phenotypic markers” of response to As-induced abiotic stress and of the Si alleviating role in ripening tomato fruit that, though complex to validate, would play a key role in studying phenomena where genetic markers alone should not suffice.

## Author Contributions

MM designed the experiment. MM, FM, DI, and GL performed the experiments and experimental measures. MM, FM, and NM analyzed and interpreted all the results. MM, FM, DI, and GL contributed to the graphical representation of data. NM commented critically on the manuscript. All the authors approved the final version of the article.

## Conflict of Interest Statement

The authors declare that the research was conducted in the absence of any commercial or financial relationships that could be construed as a potential conflict of interest.

## Footnotes

1 http://faostat.fao.org/

2 solgenomics.net

3 http://ibm.com/analytics/us/en/technology/spss/

4 www.r-project.org

## Abbreviations

ABTS: 2,2′-azinobis-(3-ethylbenzothiazoline-6-sulfonic acid)
AsA: ascorbate
β ca: β carotene
Car: total carotenoids
DHA: dehydroascorbate
DHAR: dehydroascorbate reductase
DPPH: 2,2-Diphenyl-1-picrylhydrazyl
DTNB: 5,5-dithiobis-(2-nitrobenzoic acid)
DTT: dithiothreitol
DVs: dependent variables
EC: electrical conductivity
EDTA: ethylenediaminetetraacetic acid
eq: equivalent
FA: Factor Analysis
fw: fresh weight
GA: gallic acid
GSH: glutathione
GSSG: oxidized glutathione
H_2_O_2_: hydrogen peroxide
IVs: independent variables
Lyc: lycopene
MDA: malondialdehyde
NADPH: nicotinamide adenine dinucleotide phosphate
NEM: *n*-ethylmaleimide
PBS: phosphate buffered saline
PC: Principal Component
PCA: Principal Component Analysis
ROS: reactive oxygen species
t0: control plants
t48h: 48 h treatment period
t14d: 14-days treatment period
TBA: thiobarbituric acid
TCA: trichloroacetic acid
TPs: total phenolics
