# Supplementary material for: Abiotic Stress Response to As and As+Si, Composite Reprogramming of Fruit Metabolites in Tomato Cultivars
Source: Front Plant Sci. 2017 Dec 22;8:2201. doi: 10.3389/fpls.2017.02201 (PMC5744081; doi:10.3389/fpls.2017.02201)
Supplement: Supplementary file 1 [file Data_Sheet_1.PDF]

frontiers

Supplementary Material

Abiotic stress response to As and As+Si, synergistic reprogramming of fruit metabolites in tomato cultivars

Marta Marmiroli*, Francesca Mussi, Davide Imperiale, Giacomo Lencioni, Nelson Marmiroli

***** Corresponding Author: marta.marmiroli@unipr.it

1 Supplementary Materials and Methods

Statistics

All the necessary assumptions required to perform a MANOVA were tested for this dataset, Box's M test for the homogeneity of covariance matrices proved to be non-significant, and the Bartlett test of multivariate sphericity was successfully performed (Tabachnick & Fidell 2007). The multivariate statistics considered were Wilk's Λ (lambda) and Pillai's V. The threshold for the multivariate p was set at 0.001 (equivalent to a confidence interval of 99%), and the multivariate effect size calculated as 1-Λ (1/s), following Cohen & Nee (1987) and Vacha-Haase & Thompson (2004). Three-way ANOVAs were performed after establishing normality of distribution using Levene's test on the individual dependent variables. The threshold for the univariate p was set at 0.001 and the univariate effect size was calculated following the suggestion of Fidler & Thompson (2001). *Post hoc* HSD Tukey's test was performed for each dependent variable within each independent variable (Field 2013).

For the dimension reduction analysis, two approaches were adopted, namely principal component analysis (PCA) and exploratory factor analysis (FA): the major difference between these are explained by Joliffe (1992) and Tabachnick & Fidell (2007). To ascertain the suitability of the datasets, the KMO (Kaiser-Mayer-Olkin) index was calculated; since this yielded a value >0.6, factor reduction was considered to be justifiable. The number of factors/components extracted was set by applying the Kaiser criterion using eigenvalues λ >1. Eigenvalues and number of extracted dimensions were also verified by applying Multiple Assignation Method using rawpar.sps software (O'Connor 2000; https://people.ok.ubc.ca/brioconn/nfactors/nfactors.html).

References

Cohen, J., and Nee, J. C. (1987). A comparison of two noncentral F approximations, with applications to power analysis in set correlation. *Multivariate Behav. Res.* 22, 483–490. doi:10.1207/s15327906mbr2204.

Fidler, F., and Thompson, B. (2001). Computing Correct Confidence Intervals for Anova Fixed-and Random-Effects Effect Sizes. *Educ. Psychol. Meas.* 61, 575–604. doi:10.1177/0013164401614003.

Field, A. (2013). Discovering Statistics Using IBM SPSS Statistics, The effects of brief mindfulness intervention on acute pain experience: An examination of individual difference. doi:10.1017/CBO9781107415324.004.

Joliffe, I. T., and Morgan, B. J. (1992). Principal component analysis and exploratory factor analysis. *Stat. Methods Med. Res.* 1, 69–95. doi:10.1002/9780470987605.ch14.

O'Connor, B. P. (2000). SPSS and SAS programs for determining the number of components using parallel analysis and velicer's MAP test. *Behav. Res. Methods. Instrum. Comput.* 32, 396–402. doi:10.3758/BF03200807.

Tabachnick, B. G., Fidell, L. S. (2006). Using multivariate statistics. Pearson/Al. ed. Boston. doi:10.1037/022267.

Vacha-Haase, T., and Thompson, B. (2004). How to Estimate and Interpret Various Effect Sizes. *J. Couns. Psychol.* 51, 473–481. doi:10.1037/0022-0167.51.4.473.

2 Supplementary Figures and Tables

Table S1. Three-ways ANOVA for fruits of tomato cultivars Aragon and Gladis.

Main Effect and interactions	Dependent variables	F	Signif.	Effect size η^2 (%)
**Cultivar**	ABTS (% inhibition mg^{-1} fw)	164.869	***	84.2
	DPPH (% inhibition mg^{-1} fw)	820.063	***	96.4
	TP (µg GA eq g^{-1} fw)	237.637	***	88.5
	G Redox state	460.771	***	93.7
	AsA redox state	536.374	***	94.5
	H_2O_2 nmol g^{-1} fw	244.071	***	88.7
	MDA µM g^{-1} fw	46.811	***	60.2
	Lyc (µg g^{-1})	.002	ns	0
	Car (µg β-Ca eq g^{-1} fw)	9.575	**	23.6
	As (µg g^{-1})	814.982	***	96.3
	Si (mg Kg^{-1})	88.166	***	74.0
**Treatment**	ABTS (% inhibition mg^{-1} fw)	11.527	***	42.7
	DPPH (% inhibition mg^{-1} fw)	61.208	***	79.8
	TP (µg GA eq g^{-1} fw)	6.038	**	28.0
	G Redox state	3.225	ns	17.2
	AsA redox state	167.080	***	91.5
	H_2O_2 nmol g^{-1} fw	56.814	***	78.6
	MDA µM g^{-1} fw	3.091	ns	16.6
	Lyc (µg g^{-1})	120.120	***	88.6
	Car (µg β-Ca eq g^{-1} fw)	59.242	***	79.3
	As (µg g^{-1})	537.472	***	97.2
	Si (mg Kg^{-1})	59.933	***	795
	ABTS (% inhibition mg^{-1} fw)	7.262	**	31.9

	DPPH (% inhibition mg^{-1} fw)	94.371	***	85.9
	TP (µg GA eq g^{-1} fw)	16.059	***	50.9
	G Redox state	40.022	***	72.1
	AsA redox state	44.281	***	74.1
**Time**	H_2O_2 nmol g^{-1} fw	180.480	***	92.1
	MDA µM g^{-1} fw	6.407	**	29.2
	Lyc (µg g^{-1})	245.502	***	94.1
	Car (µg β-Ca eq g^{-1} fw)	60.373	***	79.6
	As (µg g^{-1})	1869.015	***	99.2
	Si (mg Kg^{-1})	548.809	***	97.3
	ABTS (% inhibition mg^{-1} fw)	4.248	*	21.5
	DPPH (% inhibition mg^{-1} fw)	24.359	***	61.1
	TP (µg GA eq g^{-1} fw)	117.549	***	88.4
	G Redox state	2.003	ns	11.4
	AsA redox state	61.045	***	79.8
**Cultivar * Treatment**	H_2O_2 nmol g^{-1} fw	32.341	***	67.6
	MDA µM g^{-1} fw	21.996	***	58.7
	Lyc (µg g^{-1})	.261	ns	01.7
	Car (µg β-Ca eq g^{-1} fw)	5.141	*	24.9
	As (µg g^{-1})	312.145	***	95.3
	Si (mg Kg^{-1})	39.232	***	71.7
	ABTS (% inhibition mg^{-1} fw)	16.667	***	51.8
	DPPH (% inhibition mg^{-1} fw)	33.466	***	68.3
	TP (µg GA eq g^{-1} fw)	78.849	***	83.6
	G Redox state	90.484	***	85.4
**Cultivar * Time**	AsA redox state	37.263	***	70.6
	H_2O_2 nmol g^{-1} fw	101.325	***	86.7
	MDA µM g^{-1} fw	21.323	***	57.9
	Lyc (µg g^{-1})	25.121	***	61.8
	Car (µg β-Ca eq g^{-1} fw)	3.066	ns	16.5

	As (µg g^{-1})	664.137	***	97.7
	Si (mg Kg^{-1})	1.236	ns	07.4
**Treatment * Time**	ABTS (% inhibition mg^{-1} fw)	5.046	**	39.4
	DPPH (% inhibition mg^{-1} fw)	19.045	***	71.1
	TP (µg GA eq g^{-1} fw)	12.318	***	61.4
	G Redox state	7.516	***	49.2
	AsA redox state	48.106	***	86.1
	H_2O_2 nmol g^{-1} fw	34.079	***	81.5
	MDA µM g^{-1} fw	5.078	**	39.6
	Lyc (µg g^{-1})	86.889	***	91.8
	Car (µg β-Ca eq g^{-1} fw)	19.965	***	72.0
	As (µg g^{-1})	487.186	***	98.4
	Si (mg Kg^{-1})	19.142	***	71.2
**Cultivar * Treatment * Time**	ABTS (% inhibition mg^{-1} fw)	1.392	ns	15.2
	DPPH (% inhibition mg^{-1} fw)	6.652	***	46.2
	TP (µg GA eq g^{-1} fw)	40.094	***	83.8
	G Redox state	26.329	***	77.3
	AsA redox state	18.497	***	70.5
	H_2O_2 nmol g^{-1} fw	48.255	***	86.2
	MDA µM g^{-1} fw	9.748	***	55.7
	Lyc (µg g^{-1})	7.514	***	492
	Car (µg β-Ca eq g^{-1} fw)	8.443	***	52.1
	As (µg g^{-1})	254.525	***	97.0
	Si (mg Kg^{-1})	11.654	***	60.1

All ANOVA assumptions have been verified, Levene’s test is not significant. F= F test; Signif.= significance (*: $p \leq 0.05$; **: $p \leq 0.01$; ***: $p \leq 0.001$; ns= not significant); η^2 = univariate effect size (%).

Table S2. Principal component Analysis (PCA) Orthogonally Rotated Component Matrix and Factor Analysis (FA) Obliquely Rotated Pattern Matrix for fruits of both cultivars.

**Variable**	**Component**			**Factor**		
	**1**	**2**	**3**	**1**	**2**	**3**
ABTS (% inhibition mg^{-1} fw)	.926	-.131	.099	.918	-.103	.147
DPPH (% inhibition mg^{-1} fw)	.925	.218	-.043	.957	.224	-.054
TP (µg GA eq g^{-1} fw)	.818	-.231	-.048	.762	-.192	.021
H_2O_2 nM g^{-1} fw	-.717	-.182	.094	-.641	-.142	.043
G Redox state	.629	.134	.608	.545	.216	.640
Carotenoids (µg β-Ca eq g^{-1} fw)	.026	.950	-.036	.042	.969	-.054
Lycopene (µg g^{-1})	.007	.900	.158	-.003	.874	.150
AsA redox state	-.584	.585	-.298	-.520	.487	-.345
MDA (µM g^{-1} fw)	-.441	.233	.629	-.426	.216	.439
Si (mg kg^{-1})	.136	-.072	.697	.083	-.023	.439
As (µg g^{-1} As)	.280	.549	-.565	.325	.425	-.570
Eigenvalue λ	3.95	2.60	1.66	3.96	2.61	1.65
% variance explained (total or shared)	36.0	23.7	15.0	33.7	21.8	10.8
% of total variance explained		74.7				
% of shared variance explained					66.3	

2.1 Supplementary Figures

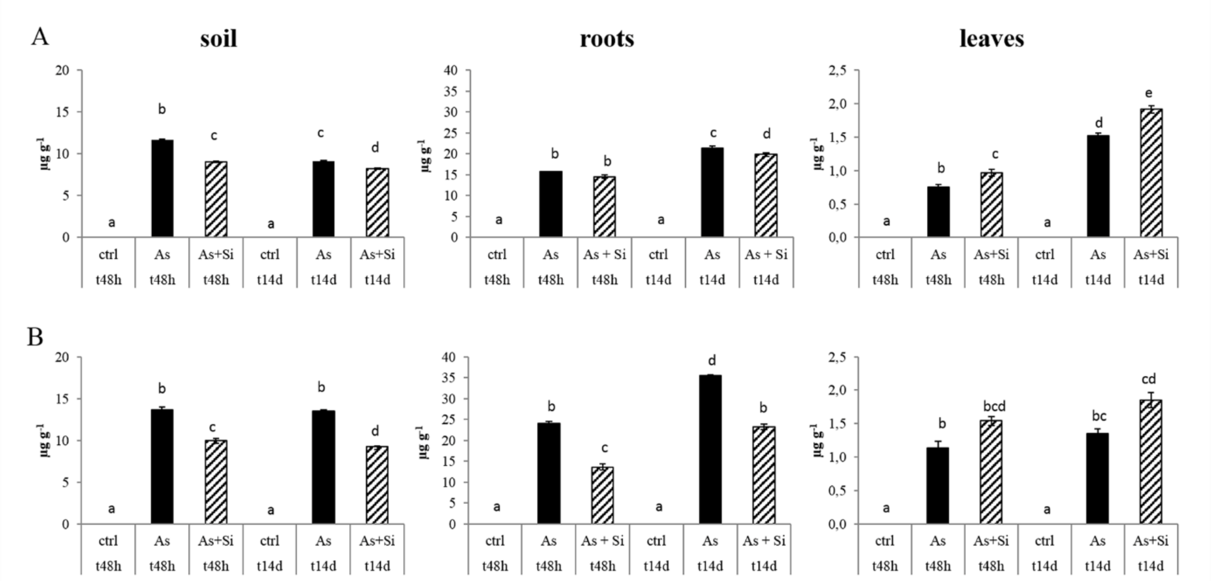

Supplementary Figure S1. Arsenic content in soil, roots and leaves of Aragon (A) and Gladis (B). Different superscript alphabet histograms are significantly different according to ANOVA followed by post-hoc Tukey's HSD test for multiple comparisons analysis ($p \leq 0.01$). Values equal to 0 means below detection limit (BDL).

Figure S2

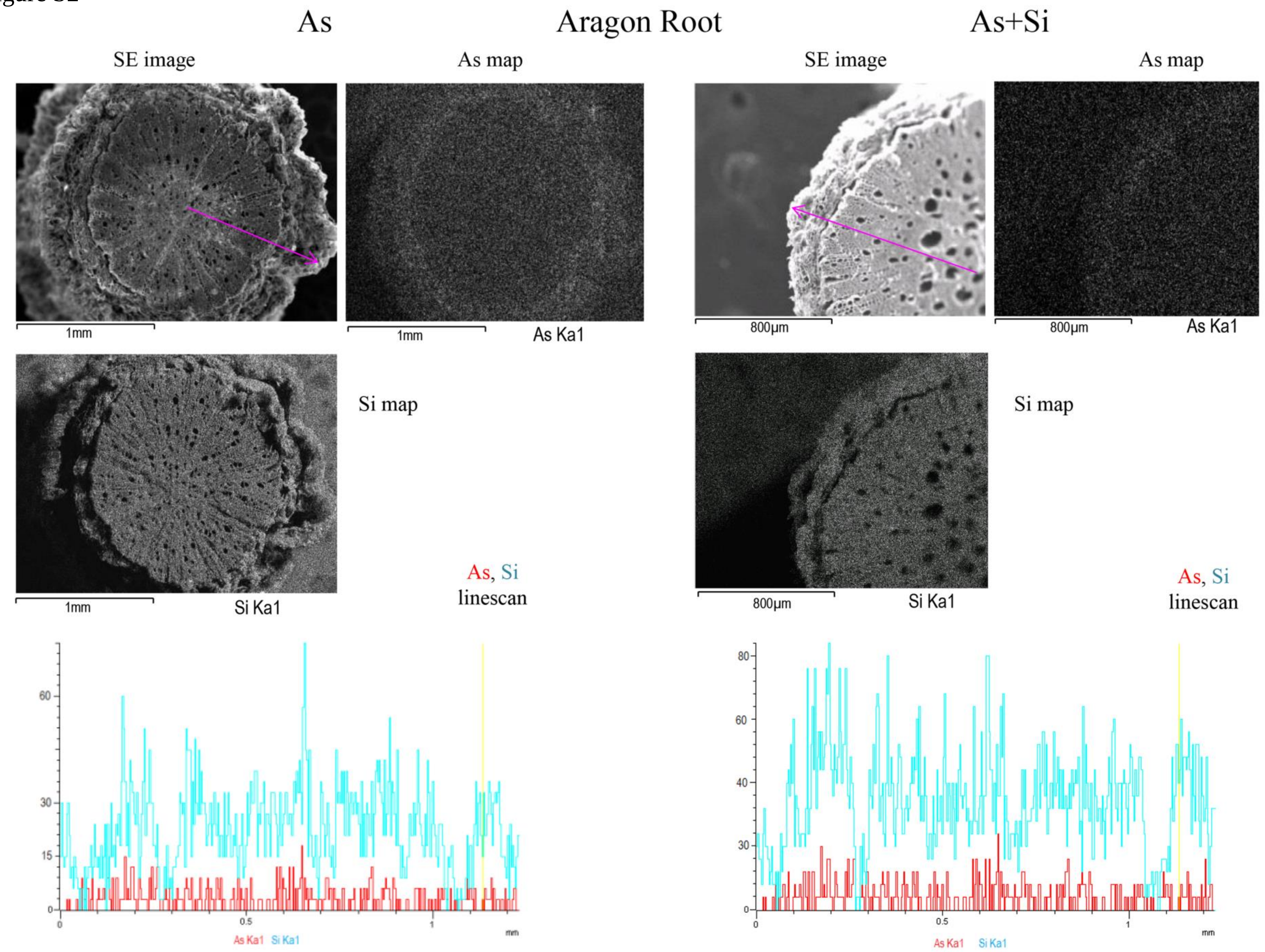
As
Aragon Root
As+Si
SE image
As map
SE image
As map
1mm
1mm
As Ka1
800µm
800µm
As Ka1
Si map
Si map
1mm
Si Ka1
800µm
Si Ka1
As, Si
linescan
As, Si
linescan
As Ka1 Si Ka1
As Ka1 Si Ka1

Supplementary Figure S2: SEM/EDX As and Si root analysis. Secondary Electrons (SE) image, As (Kα1) emission X-ray dot-map, As (Kα1) and Si (Kα1) line-scans for root of Aragon under As (left) and As+Si (right) treatments. The Y axis in line-scans measure X-ray cps during the whole line-scan acquisition, the X axis corresponds to the scanned line (drawn in pink on the SE image). In SE images black bar at bottom left indicates cross section size. In dot-maps lighter shades of gray indicate higher concentrations of As or Si, in line-scans red is for As, blue is for Si.

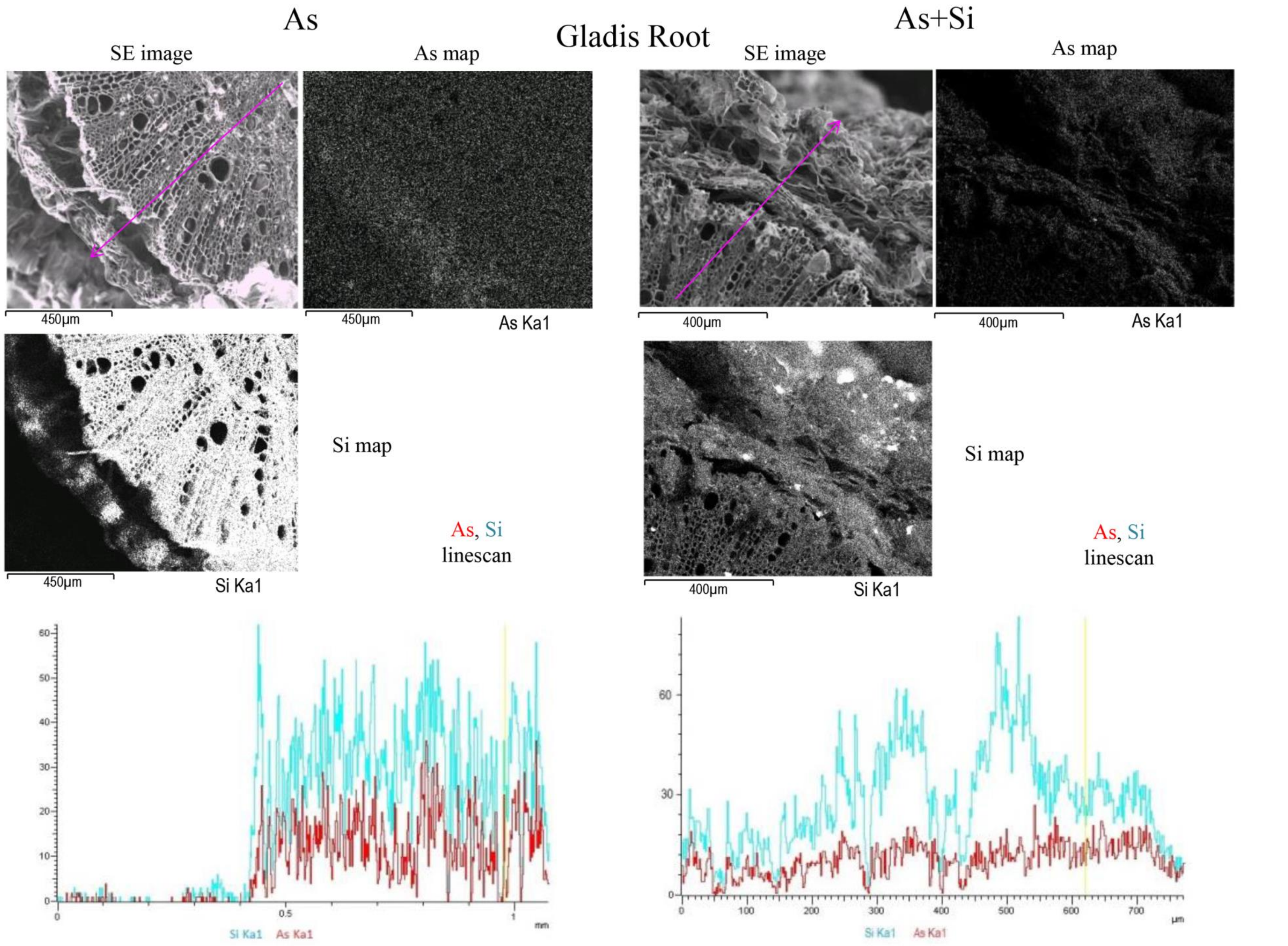
As
Gladis Root
As+Si
SE image
As map
SE image
As map
450µm
450µm
As Ka1
400µm
400µm
As Ka1
Si map
Si map
450µm
Si Ka1
400µm
Si Ka1
As, Si
linescan
As, Si
linescan
Si Ka1 As Ka1
Si Ka1 As Ka1

Figure S3B

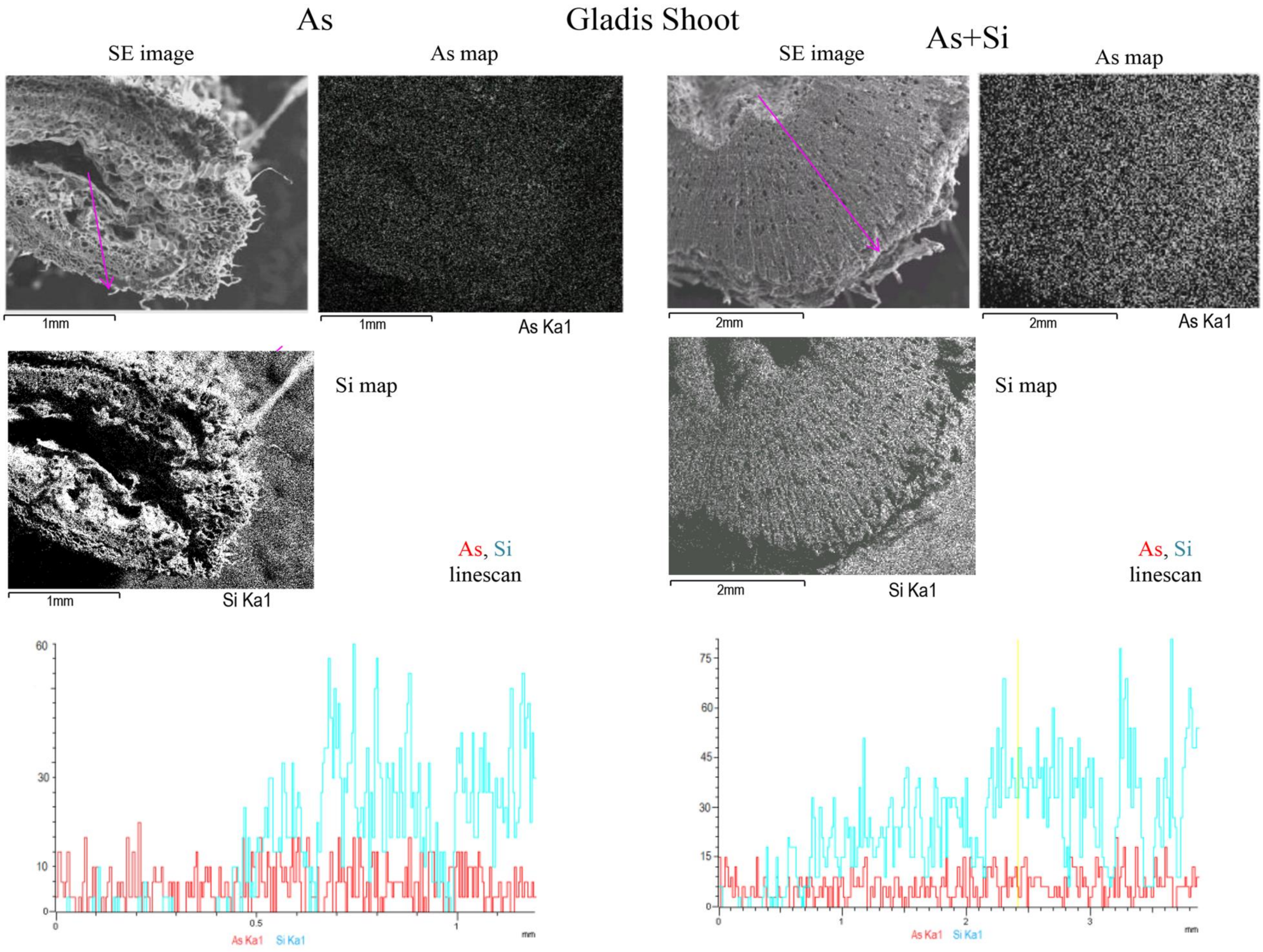

As
Gladis Shoot
As+Si
SE image
As map
1mm
As Ka1
Si map
As, Si
linescan
Si Ka1
2mm
As Ka1 Si Ka1
mm

Supplementary Figure S3: SEM/EDX As and Si root and shoot analysis. Secondary Electrons (SE) image, As (Kα1) emission X-ray dot-map, As (Kα1) and Si (Kα1) line-scans for root (A) and shoot (B) of Gladis under As (left) and As+Si (right) treatments. The Y axis in line-scans measure X-ray cps during the whole line-scan acquisition, the X axis corresponds to the scanned line (drawn in pink on the SE image). In SE images black bar at bottom left indicates cross section size. In dot-maps lighter shades of gray indicate higher concentrations of As or Si, in line-scans red is for As, blue is for Si.

Figure S4A

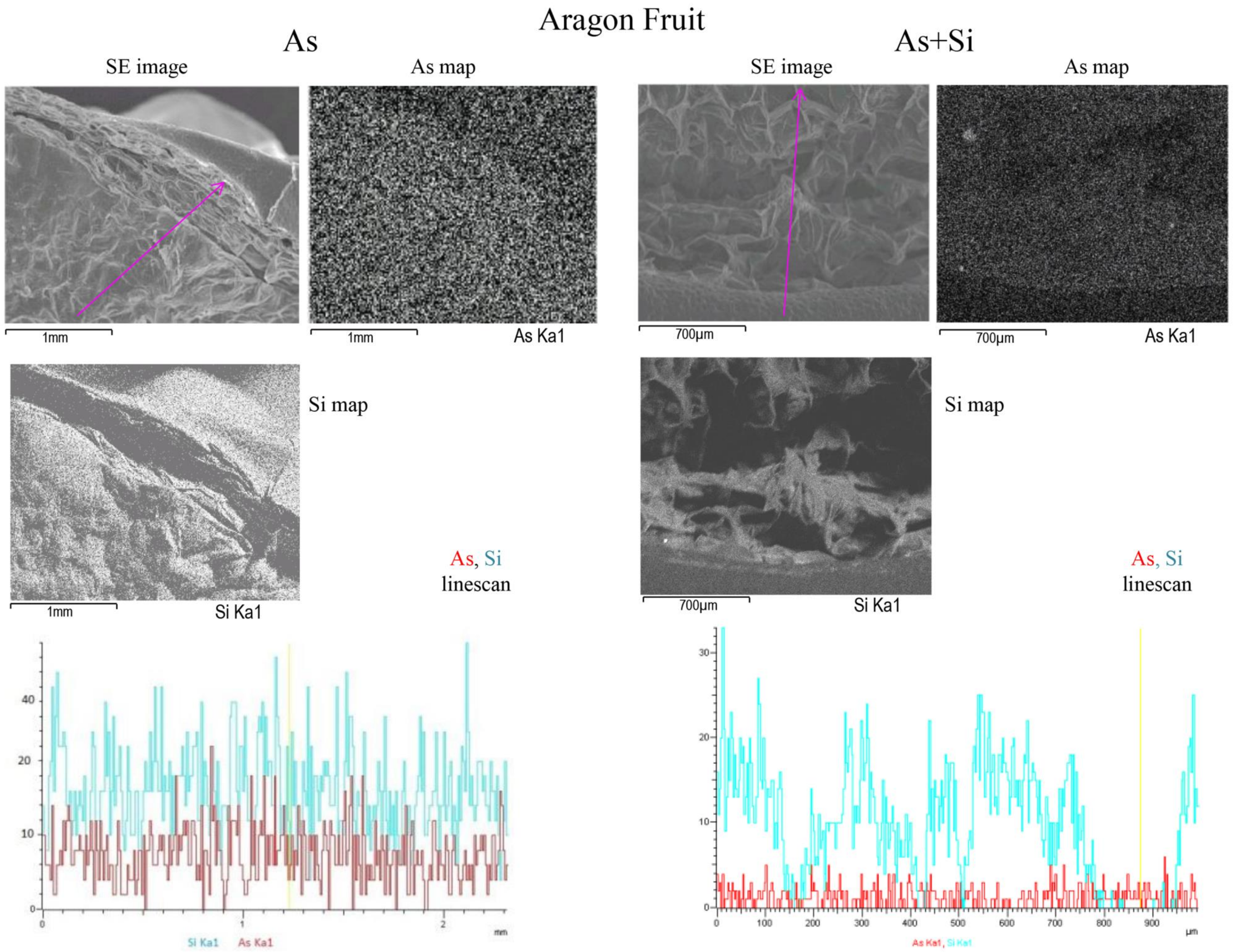

Aragon Fruit
As
As+Si
SE image
As map
SE image
As map
1mm
1mm
As Ka1
700µm
700µm
As Ka1
Si map
Si map
1mm
Si Ka1
700µm
Si Ka1
As, Si
linescan
As, Si
linescan
Si Ka1
As Ka1
mm
µm
As Ka1, Si Ka1

Figure S4B

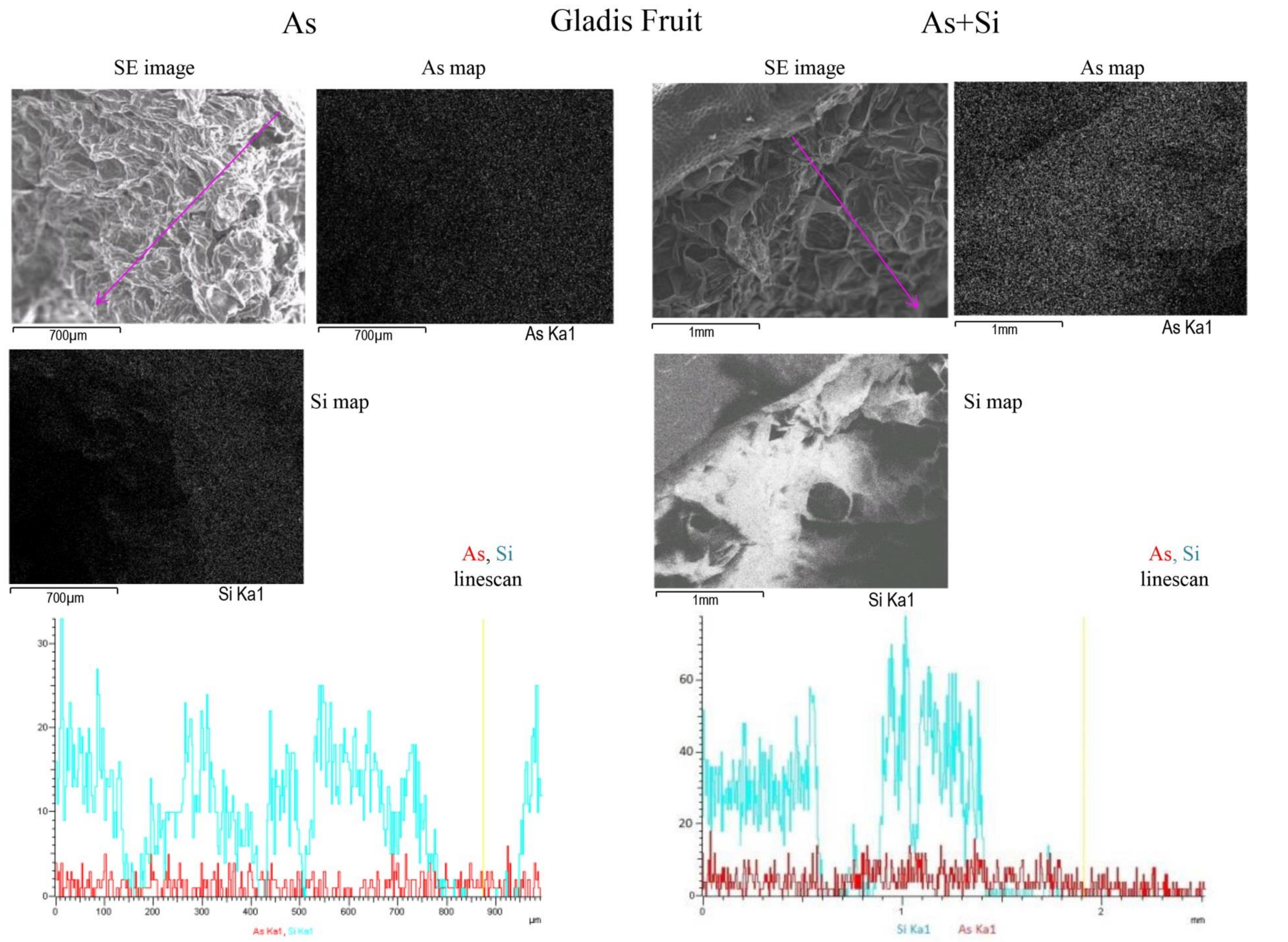
As
Gladis Fruit
As+Si
SE image
As map
SE image
As map
700µm
700µm
As Ka1
1mm
1mm
As Ka1
Si map
Si map
As, Si
linescan
As, Si
linescan
700µm
Si Ka1
1mm
Si Ka1
µm
mm
As Ka1, Si Ka1
Si Ka1
As Ka1

Supplementary Figure S4: SEM/EDX As and Si fruit analysis. Fruit Secondary Electrons (SE) image, As (Kα1) emission X-ray dot-map, As (Kα1) and Si (Kα1) line-scans of Aragon (A) and Gladis (B) under As (left) and As+Si (right) treatments. The Y axis in line-scans measure X-ray cps during the whole line-scan acquisition, the X axis corresponds to the scanned line (drawn in pink on the SE image). In SE images black bar at bottom left indicates cross section size. In dot-maps lighter shades of gray indicate higher concentrations of As or Si, in line-scans red is for As, blue is for Si.

Figure S5

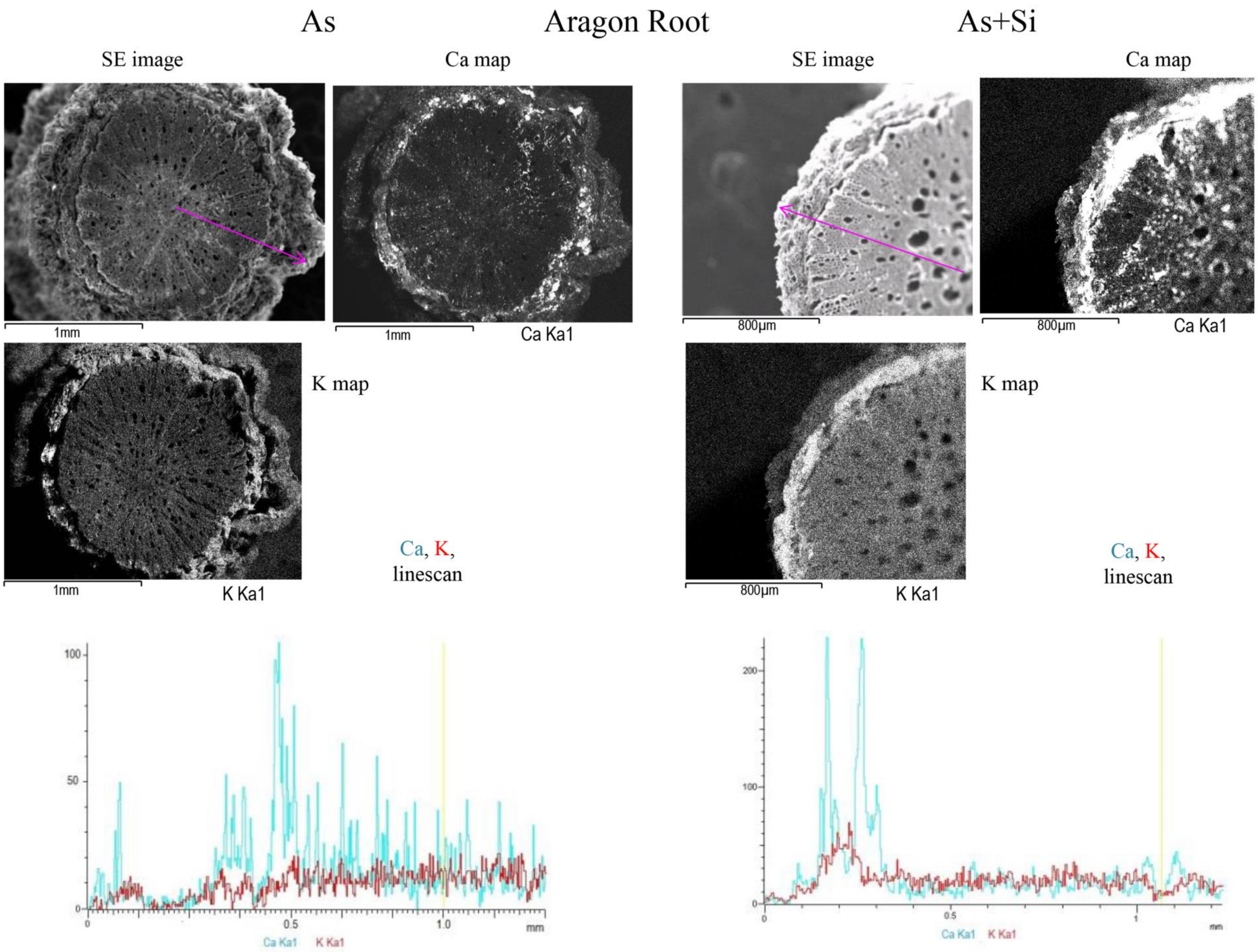
As
Aragon Root
As+Si
SE image
Ca map
SE image
Ca map
1mm
1mm
Ca Ka1
800µm
800µm
Ca Ka1
K map
K map
Ca, K,
linescan
Ca, K,
linescan
1mm
K Ka1
800µm
K Ka1
Ca Ka1
K Ka1
Ca Ka1
K Ka1
mm
mm

Supplementary Figure S5: SEM/EDX Ca and K root, shoot, and fruit analysis. Secondary electrons (SE) image, Ca (Kα1) and K (Kα1) line-scans for root (top), shoot (middle), and fruit (bottom) of Gladis under As (left) and As+Si (right) treatments. The Y axis in line-scans measure X-ray cps during the whole line-scan acquisition, the X axis corresponds to the scanned line (drawn in pink on the SE image). In SE images black bar at bottom left indicates cross section size, in line-scans red is for K, blue is for Ca.

Figure S6

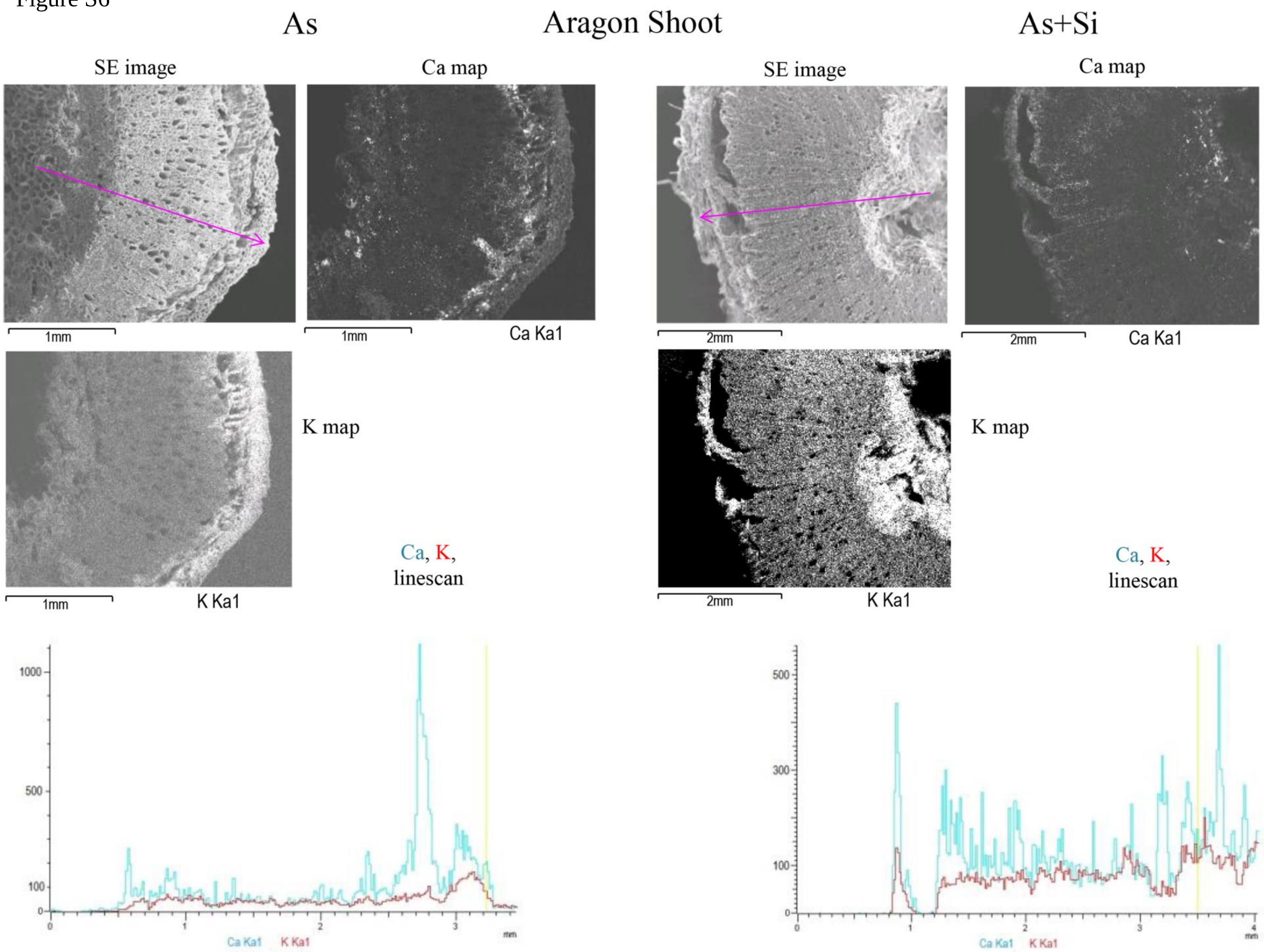
As
Aragon Shoot
As+Si
SE image
Ca map
SE image
Ca map
1mm
1mm
Ca Ka1
2mm
2mm
Ca Ka1
K map
K map
Ca, K,
linescan
Ca, K,
linescan
1mm
K Ka1
2mm
K Ka1
1000
500
100
0
0
1
2
3
mm
Ca Ka1
K Ka1
500
300
100
0
0
1
2
3
4
mm
Ca Ka1
K Ka1

Supplementary Figure S6: SEM/EDX Ca and K shoot analysis. Secondary Electrons (SE) image, As (Kα1) emission X-ray dot-map, Ca (Kα1) and K (Kα1) line-scans for shoot of Aragon under As (left) and As+Si (right) treatments. The Y axis in line-scans measure X-ray cps during the whole line-scan acquisition, the X axis corresponds to the scanned line (drawn in pink on the SE image). In SE images black bar at bottom left indicates cross section size. In dot-maps lighter shades of gray indicate higher concentrations of Ca or K, in line-scans red is for K, blue is for Ca.

Figure S7

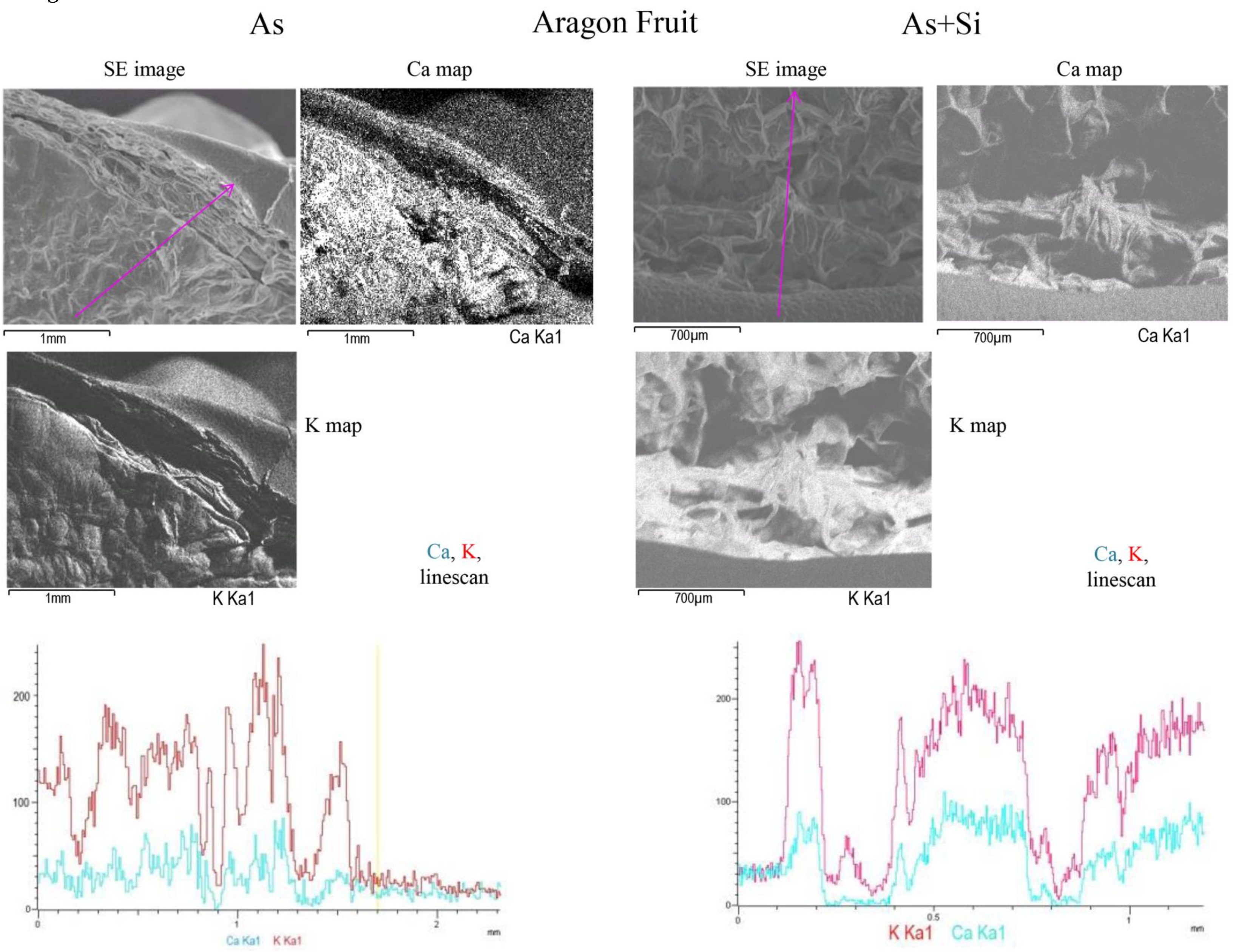
As
Aragon Fruit
As+Si
SE image
Ca map
SE image
Ca map
1mm
1mm
Ca Ka1
700µm
700µm
Ca Ka1
K map
K map
Ca, K,
linescan
Ca, K,
linescan
1mm
K Ka1
700µm
K Ka1
200
100
0
0
1
2
mm
Ca Ka1 K Ka1
200
100
0
0
0.5
1
mm
K Ka1 Ca Ka1

Supplementary Figure S7: SEM/EDX Ca and K fruit analysis. Secondary Electrons (SE) image, As (Kα1) emission X-ray dot-map, Ca (Kα1) and K (Kα1) line-scans for fruit of Aragon under As (left) and As+Si (right) treatments. The Y axis in line-scans measure X-ray cps during the whole line-scan acquisition, the X axis corresponds to the scanned line (drawn in pink on the SE image). In SE images black bar at bottom left indicates cross section size. In dot-maps lighter shades of gray indicate higher concentrations of Ca or K, in line-scans red is for K, blue is for Ca.

Figure S8

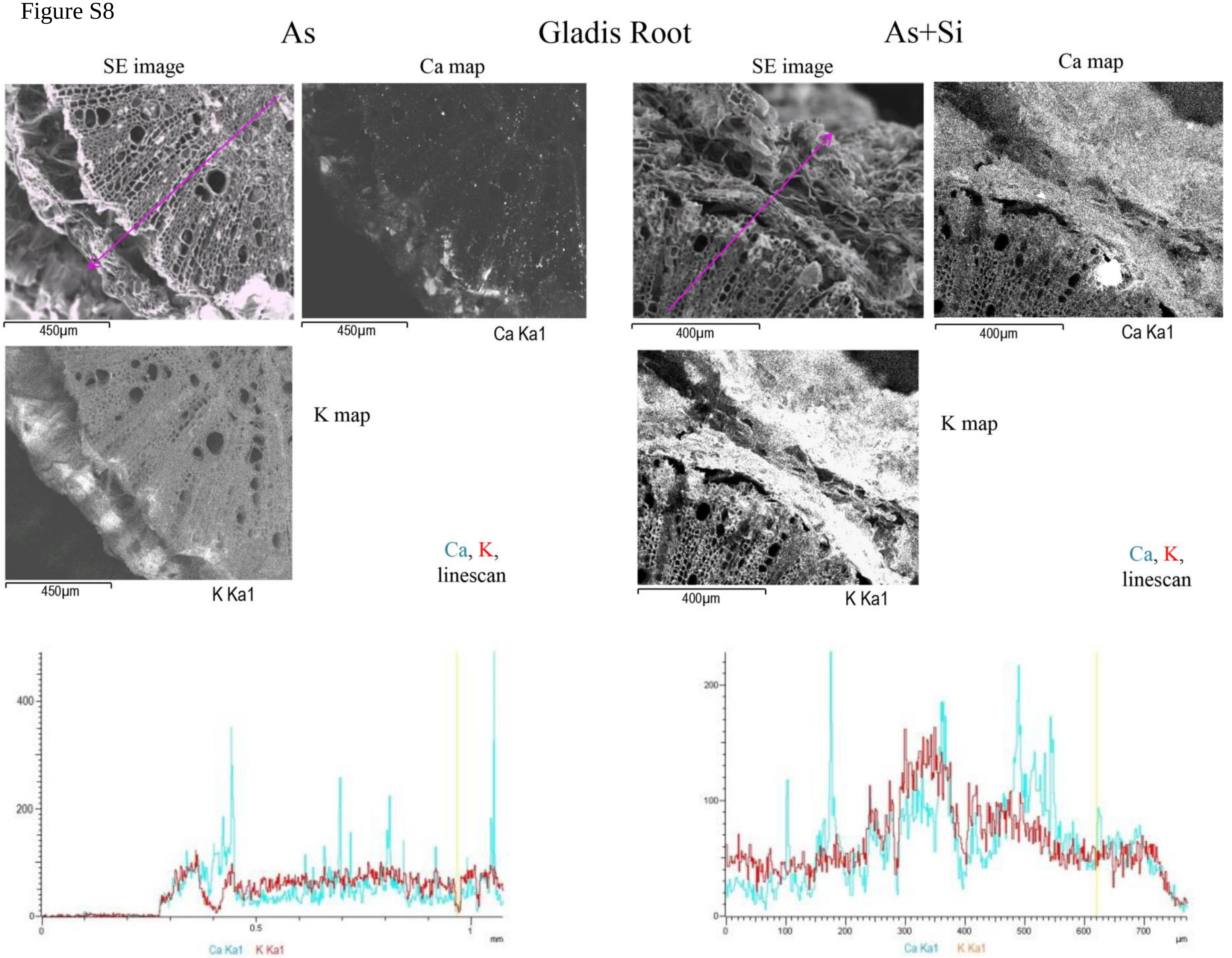
As
Gladis Root
As+Si
SE image
Ca map
SE image
Ca map
450µm
450µm
Ca Ka1
400µm
400µm
Ca Ka1
K map
K map
Ca, K,
linescan
Ca, K,
linescan
450µm
K Ka1
400µm
K Ka1
Ca Ka1
K Ka1
Ca Ka1
K Ka1

Supplementary Figure S8: SEM/EDX Ca and K root analysis. Secondary Electrons (SE) image, As (Kα1) emission X-ray dot-map, Ca (Kα1) and K (Kα1) line-scans for roots of Gladis under As (left) and As+Si (right) treatments. The Y axis in line-scans measure X-ray cps during the whole line-scan acquisition, the X axis corresponds to the scanned line (drawn in pink on the SE image). In SE images black bar at bottom left indicates cross section size. In dot-maps lighter shades of gray indicate higher concentrations of Ca or K, in line-scans red is for K, blue is for Ca.

Figure S9

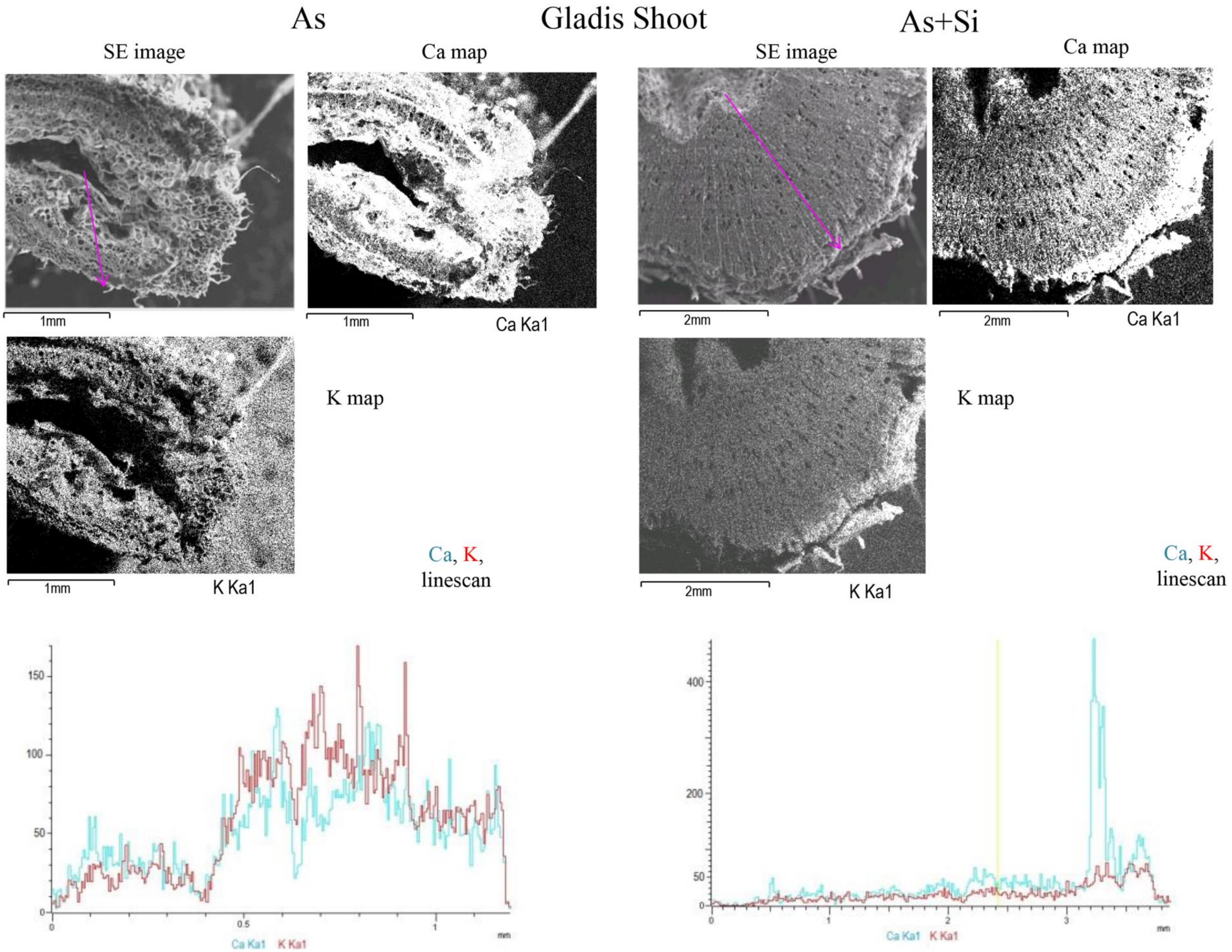
As
Gladis Shoot
As+Si
SE image
Ca map
SE image
Ca map
1mm
1mm
Ca Ka1
2mm
2mm
Ca Ka1
K map
K map
1mm
K Ka1
Ca, K, linescan
2mm
K Ka1
Ca, K, linescan
Ca Ka1 K Ka1
Ca Ka1 K Ka1

Supplementary Figure S9: SEM/EDX Ca and K shoot analysis. Secondary Electrons (SE) image, As (Kα1) emission X-ray dot-map, Ca (Kα1) and K (Kα1) line-scans for shoot of Gladis under As (left) and As+Si (right) treatments. The Y axis in line-scans measure X-ray cps during the whole line-scan acquisition, the X axis corresponds to the scanned line (drawn in pink on the SE image). In SE images black bar at bottom left indicates cross section size. In dot-maps lighter shades of gray indicate higher concentrations of Ca or K, in line-scans red is for K, blue is for Ca.

Figure S10

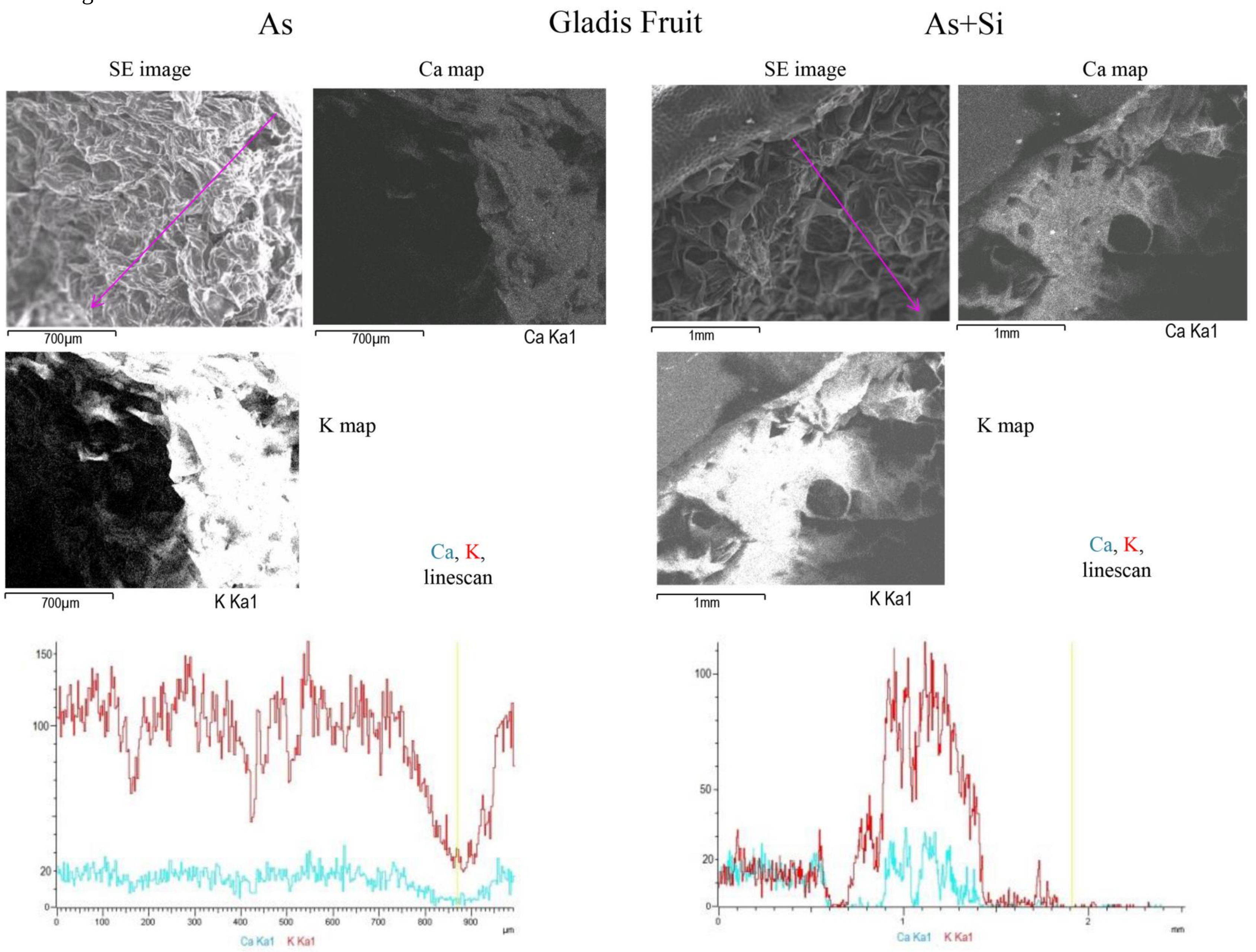
As
Gladis Fruit
As+Si
SE image
Ca map
SE image
Ca map
700µm
700µm
Ca Ka1
1mm
1mm
Ca Ka1
K map
K map
Ca, K,
linescan
Ca, K,
linescan
700µm
K Ka1
1mm
K Ka1
Ca Ka1 K Ka1
Ca Ka1 K Ka1
µm
mm

Supplementary Figure S10: SEM/EDX Ca and K fruit analysis. Secondary Electrons (SE) image, As (Kα1) emission X-ray dot-map, Ca (Kα1) and K (Kα1) line-scans for fruit of Gladis under As (left) and As+Si (right) treatments. The Y axis in line-scans measure X-ray cps during the whole line-scan acquisition, the X axis corresponds to the scanned line (drawn in pink on the SE image). In SE images black bar at bottom left indicates cross section size. In dot-maps lighter shades of gray indicate higher concentrations of Ca or K, in line-scans red is for K, blue is for Ca.

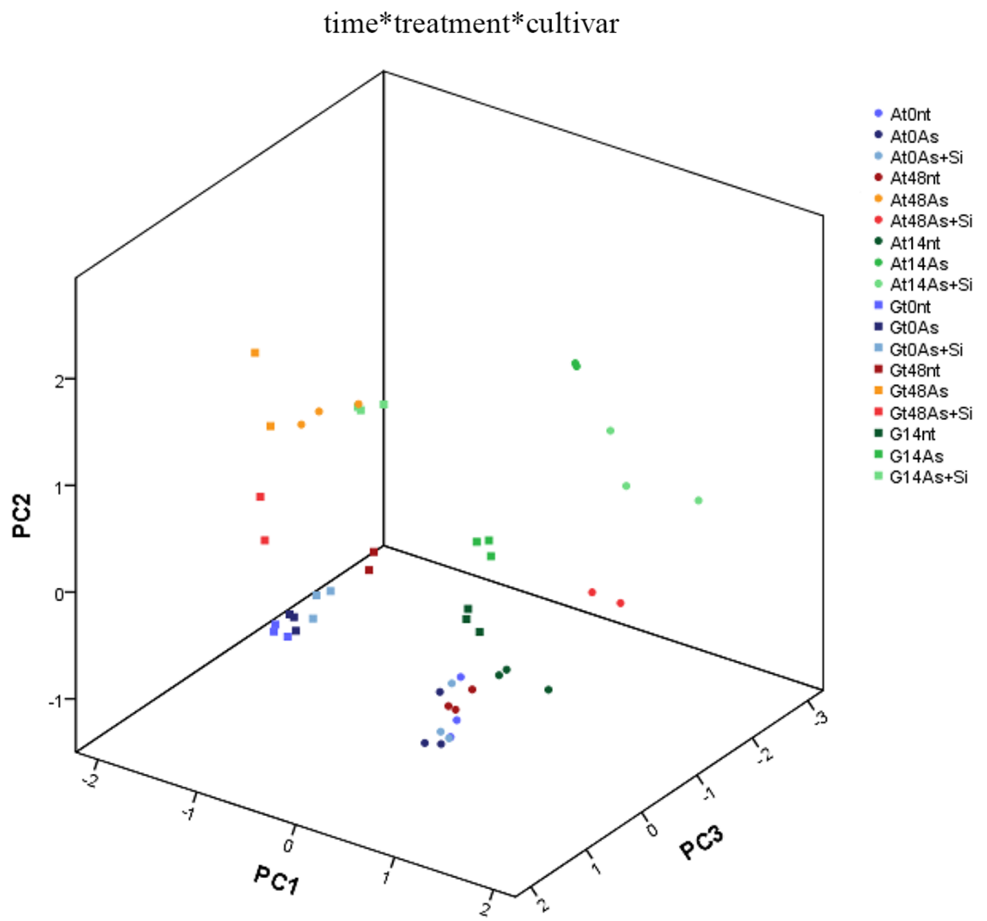

Supplementary Figure S11: PCA of the fruits, considering simultaneously the three variables cultivar, treatment and time of treatment. KMO (Kaiser-Mayer-Olkin) index = 0.67. The components extraction criteria were: eigenvalue $\lambda>1$ and varimax (orthogonal) rotation. The total proportion of the overall variance explained (e.v.) was 74.7%. PC1: $\lambda=3.9$, e.v. = 36.0%. PC2: $\lambda=2.6$, e.v. = 23.7%. PC3: $\lambda=1.7$, e.v. = 15.0%.
